# Supplementary material for: Nature exposure induces analgesic effects by acting on nociception-related neural processing
Source: Nat Commun. 2025 Mar 13;16:2037. doi: 10.1038/s41467-025-56870-2 (PMC11906725; doi:10.1038/s41467-025-56870-2)
Supplement: Supplementary file 1 — Supplementary Information [file 41467_2025_56870_MOESM1_ESM.pdf]

Supplementary Information for

Nature exposure induces analgesic effects by acting on nociception-related neural processing

Maximilian O. Steininger[1], Mathew P. White[2,3,4], Lukas Lengersdorff[1], Lei Zhang[5,6,7], Alexander J. Smalley[3], Simone Kühn[8,9], Claus Lamm[1,2,4*]

[1]Social, Cognitive and Affective Neuroscience Unit, Department of Cognition, Emotion, and Methods in Psychology, Faculty of Psychology, University of Vienna, Vienna, Austria
[2]Cognitive Science Hub, University of Vienna, Vienna, Austria
[3]European Centre for Environment and Human Health, University of Exeter, Truro, UK
[4]Environment and Climate Research Hub, University of Vienna, Vienna, Austria
[5]Centre for Human Brain Health, School of Psychology, University of Birmingham, Birmingham, UK
[6]Institute for Mental Health, School of Psychology, University of Birmingham, Birmingham, UK
[7] Centre for Developmental Science, School of Psychology, University of Birmingham, Birmingham, UK
[8]Center for Environmental Neuroscience, Max Planck Institute for Human Development, Berlin, Germany
[9]Department of Psychiatry, University Medical Center Hamburg-Eppendorf, Hamburg, Germany

*Corresponding author: Claus Lamm
Email: claus.lamm@univie.ac.at

This PDF file includes:

Supplementary Methods
Supplementary Results
Supplementary Figure 1 – 3
Supplementary Table 1 – 17
Supplementary References

Supplementary Methods

Inclusion Criteria for Participants. Participants fulfilling the following criteria were included in the study: right-handedness, no past or present neurological, psychiatric, or chronic disorder, no past or current disorder affecting the perception of pain, no intake of psychopharmacological medication (including pain medication), no substance abuse (alcohol, drugs) within the last three months, no past or present enrollment in studies including pharmaceutics, medicine or psychology, no pregnancy, age between 18 and 35 years. Furthermore, standard criteria for being able to take part in a functional magnetic resonance imaging (fMRI) experiment had to be fulfilled by the participants.

Power Analysis. We conducted an a-priori power analysis, which yielded a planned sample size of N = 41 participants. This sample size was based on a power analysis conducted for a repeated measures ANOVA model using G*Power 3.1[1]. Although the preregistered and reported statistical models in this work are linear mixed models (LMM), the project was powered for repeated measures ANOVAs, which would have served as a fallback in case of convergence issues in the LMMs[2,3]. Since, compared to repeated measures ANOVA, LMMs in most situations have a higher power, the estimated sample size from the analyses can be seen as a conservative estimate for the targeted LMMs. The power analysis was based on previous studies investigating the effect of different environmental stimuli on pain perception[4–7]. The average of reported effect sizes comparing differences between nature stimuli to a complete absence of stimulation while experiencing pain was used (Cohen's d = .65). Studies directly comparing natural to urban environments in the domain of pain research revealed an effect size of similar magnitude (Cohen's d = .71) as the average reported for the remaining studies[8]. Using a type-I error probability of α = 0.05, a power of 1-β = 0.8, an ε = 0.34 for non-sphericity corrections, and conservative estimates for repeated measures correlation of r = 0.4 between consecutive pain measurements[9–11], we estimated a targeted sample size of N = 41 participants. Because published studies may have inflated effect sizes due to publication/survivorship bias, we decided to use a larger sample size of N = 48.

Preregistration and deviations.

Generally, we strictly adhered to our preregistration, registered on 12 May 2022 on OSF (osf.io/t8dqu), transparently report deviations, and label additionally performed and exploratory analyses as such.

We deviated from the preregistration regarding the specification of our LMMs in the following points. First, most of our preregistered LMMs were specified without adhering to the principle of using maximal random effect structures. However, it is recommended to define maximal random effect structures when using LMM for confirmatory hypothesis testing, as the model outputs tend to generalize best under these circumstances[12]. Since confirmatory hypothesis testing was the intended aim of the current study, we decided to keep the random effects structure maximal when possible and justified by the design. This modification had the added benefit of overcoming convergence issues in the models we encountered using the initially specified random effect structures from the preregistration (which included only random intercept per participant). Model formulae for each conducted LMM in the main manuscript can be found in this document.

Second, concerning the LMM of the immediate pain ratings (i.e., intensity and unpleasantness of individual shocks), we preregistered a model that included ratings of both painful and non-painful shocks. However, including non-painful shocks in the model led to convergence issues and violations of the model assumptions, possibly due to limited variation in the ratings of non-painful shocks (which were, as intended, predominantly rated as 0 or 1). Thus, we adapted the model and specified it excluding non-painful shocks. We believe that excluding the non-painful stimuli from the analysis does not compromise the relevance of our replication of previous findings in self-report. First, our study's main aim was to replicate how exposure to nature affects the processing of painful stimuli. Second, previous studies investigate how nature exposure impacts the processing of painful stimuli and do not examine nor report how these relate to sensory stimuli below the pain threshold.

Third, regarding the LMM of our neuroimaging data investigating overall neural responses to pain, we deviated in terms of the dependent variable used. To examine whether nature exposure

acts on neural indicators of ‘lower-level’ or ‘higher-level’ components of pain, we preregistered an analysis using the pooled activation of various regions of interest (ROI) as a dependent variable. The main aim of this analysis was to investigate whether the nature condition impacts the overall neural response to pain clustered into two main components (i.e., sensory-discriminative vs. affective-motivational). For this analysis, we specified a LMM in which all ROI responses related to sensory-discriminative (i.e., S1, S2, pINS, SPL) or affective-motivational (i.e., aMCC, aINS, mPFC, PAG) components were used as the dependent variable simultaneously. We included environment (nature as a reference), component (sensory-discriminative as a reference), and their interaction as fixed effects. Using this specification, we expected a significant interaction effect between environment and component, indicating that our nature condition would differentially impact the pooled activity of single ROIs clustered into sensory-discriminative vs. affective-motivational components. However, considering the feedback of colleagues, revisiting the literature on the neuroimaging of pain, and upon further reflection, we adapted our analysis plan and decided to use multivoxel pattern responses instead of pooled ROI activity to disentangle nature's effect on ‘lower-level’ or ‘higher-level’ pain processing. Importantly, this amendment was performed without peeking at the data. To this end, we used the signal of two established multivoxel pain signatures, the neurologic pain signature (NPS) and the stimulus intensity independent pain signature-1 (SIIPS1), as a dependent variable (see main manuscript). Compared to using single ROI responses, these multivariate patterns differentiate between the two components of pain with increasing precision and validity and operationalize them with high levels of specificity. Although we did preregister to compare the impact of our environments on the NPS we did not preregister using SIIPS1. We believe that using a more precise and valid operationalization of our targeted constructs increased the validity and parsimony of our findings. Nevertheless, we also calculated the originally planned LMM using the pooled activity of the single ROIs as a dependent variable and the fixed effects as specified above. As for all other models, we set the random effects structure maximal with random slopes and intercepts for environment and component by participant. Using this model, we corroborated the findings of the main manuscript by showing that nature, compared to urban or indoor scenes, also reduces pain when using a different indicator of ‘lower-level’ sensory-discriminative pain processing. For details, see the Supplementary Results section.

Additionally, we would like to clarify a potentially ambiguous wording used in our preregistration. In the preregistration, we specified the following hypothesis: Viewing virtual nature stimuli leads to a downregulation of pain primarily via activity changes in either sensory-discriminative (i.e., S1, S2, SPL, pINS) or affective-motivational (i.e., aMCC, mPFC, dlPFC, aINS, PAG) pain components. We specified that our hypothesis regarding nature’s impact on which of these two neural modulatory systems are affected is non-directional in the sense that a lack of prior studies precluded predicting with confidence whether lower-level or higher-level pain processes (or both) would be reduced. The non-directionality of this hypothesis is thus related to the interaction effect of the independent variables (i.e., environment*component). This interaction effect was also tested two-sidedly in our analysis. However, the pairwise planned contrasts that investigated whether each component separately showed a reduced response (as indicated by the wording downregulation of pain in the preregistration) were directional, which is why they were assessed with one-sided testing.

Furthermore, to ensure the robustness of our MRI analyses, we conducted several sensitivity analyses across different analytical approaches. Specifically, we reanalyzed the data by: (1) excluding participants identified as statistical outliers, (2) applying a motion threshold of 2 mm, (3) using a more parsimonious first-level model, and (4) employing alternative masks for our ROIs. Detailed descriptions of the results are provided in the Supplementary Results section Sensitivity analysis. Notably, despite these variations in analytical approaches, results for the NPS and key ROIs, including the thalamus, S2, and pINS remained consistent. However, greater variability in the outcomes was observed for the SPL and aINS, suggesting that findings in these regions should be interpreted with caution.

Regarding points (1) and (2), the primary analyses presented in the main manuscript do not exclude statistical outliers or participants exceeding motion thresholds. Although we initially planned to exclude these cases, we reconsidered this approach (before performing any analyses or looking at any data) because outliers may capture meaningful individual variability and exclusions could compromise ecological validity. Moreover, residual movement was modeled as a

nuisance regressor, and visual inspection of the data (orthogonal to any hypotheses, pain>no-pain) revealed that participants exceeding the 2mm movement criterion showed no major signs of movement artifacts. These considerations are particularly pertinent when analyzing multivariate patterns such as the NPS and SIIPS1. Upon reviewing the distribution of these pattern responses in prior research[13,14], we concluded that deviating values represent valid variability within our sample. Consequently, we opted to retain these data in our analysis. However, for full transparency and completeness of reporting all analyses, we present the results of analyses excluding these participants in the section Sensitivity analyses below.

Regarding point (3), we initially employed a parsimonious first-level model to reduce the risk of overfitting. This model included eight regressors: delivery of painful shocks, delivery of non-painful shocks, and six motion regressors. This approach was also outlined and reported in the preprint version of this article. Following reviewer feedback and in alignment with standard modeling approaches used in similar research[13,15], we revised the first-level model to incorporate three additional regressors. This revised more comprehensive model includes the following 11 regressors: anticipation of painful shocks, anticipation of non-painful shocks, delivery of painful shocks, delivery of non-painful shocks, ratings, and six motion regressors. This revised approach is reported in the main article, while the initial parsimonious model is detailed in the section Sensitivity analyses below.

Regarding point (4), the main article reports results based on ROIs that include only voxels showing a significant response to painful versus non-painful stimuli in the pain>no-pain contrast across all environments. Following a reviewer's suggestion, we reanalyzed the data using the full ROI masks that encompassed all voxels irrespective of their sensitivity to pain. These additional analyses are detailed in the section Sensitivity analyses below.

Lastly, three additional hypotheses had been part of our preregistration. These hypotheses focused on the impact of the different environments on positive and negative affect, pulse rate, and the potential moderating role of individual differences in nature-connectedness on the effects of nature. The results of these analyses and their interpretations are documented in the Supplementary Results section titled Additional preregistered analyses. Notably, we observed significant differences in positive and negative affect between the environments, a reduction in pulse rate when comparing the nature and urban environments, and no evidence of moderation effects related to nature connectedness. Furthermore, prompted by reviewer feedback, we also report non-preregistered exploratory analyses in the results section titled Additional non-preregistered analyses.

Supplementary Results

Self-reported pain. The following section reports all details regarding the LMM of the self-reported pain data for immediate (i.e., intensity and unpleasantness) as well as recollected (i.e., distraction and tolerance) ratings (see Supplementary Table 1 and 2; note that all fixed effects in the tables were tested two-sided. The fixed effects reflect comparisons relative to the reference category of each independent (categorical) variable. These comparisons generally do not align with our a priori hypotheses of interest, which are instead examined through planned pairwise comparisons based on the marginal means of the models. This approach was also consistently applied to the analyses presented in Supplementary Tables 5-16.).

Regarding the immediate ratings, we calculated a LMM specifying the ratings of the painful shocks as the dependent variable (i.e., intensity and unpleasantness) to be predicted by the fixed effect of environment (nature as the reference), rating content (intensity as the reference), and their interaction (with random slopes and intercepts for environment, rating content and their interaction by participant). We observed significant main effects of environment [$F_{(2,48.14)} = 12.49$, $p < .001$)], rating content [$F_{(1,48.00)} = 17.52$, $p < .001$)], and an interaction of environment*content [$F_{(2,81.11)} = 9.19$, $p < .001$)]. The main effect of environment and its interaction with rating content, as well as the planned pairwise contrasts, are presented in the main manuscript. The planned pairwise comparisons in the main manuscript were a priori not planned to be corrected for multiple comparisons, as they were based on assessing separate hypotheses. Notably, however, even when applying Bonferroni-Holm corrections for each dependent variable, the comparisons remained significant: nature vs. urban ($p = .037$) or indoor ($p = .015$) for intensity ratings, and nature

vs. urban ($p = .000005$) or indoor ($p = .00007$) for unpleasantness ratings. The planned pairwise contrasts for the main effect of rating content indicated that intensity ($M = 5.65$, $SE = 0.12$) was rated higher than unpleasantness ($M = 5.16$, $SE = 0.16$), irrespective of the environment shown [$b = 0.49$, $SE = 0.12$, $t_{(48)} = 4.18$, $p < .001$].

Regarding the retrospective ratings, participants were asked to give an overall assessment of how much viewing each respective environment helped them to tolerate better and distract themselves from the pain using two separate questions. This assessment was given at the end of each pain block, i.e., after watching one environment while receiving the electrical shocks. Participants answered the following two questions using a scale from one ("not at all") to five ("very"): "Sitting in [nature / the city / the room] distracted me from the electrical shocks." and "Sitting in [nature / the city / the room] helped me to better tolerate the electrical shocks.". We used this wording as a way to explicitly prompt participants to envision themselves in the respective environment before starting each pain block using a structured script. To analyze the data, we used a LMM (see Supplementary Table S2). We specified the recollected ratings (i.e., distraction and tolerance) as the dependent variable to be predicted by the fixed effect of environment (nature as the reference), rating content (distraction as the reference), and their interaction (with random slopes and intercepts for environment by participant). The significance tests for the main effects and their interaction revealed that there was no significant overall effect of environment [$F_{(2,156.75)} = 0.83$, $p = .437$)], but a significant main effect of rating content [$F_{(1,144.00)} = 10.69$, $p = .001$)], and a trend-effect for its interaction with environment [$F_{(2,144.00)} = 2.92$, $p = .056$)]. Post-hoc pairwise comparisons revealed that ratings of distraction ($M = 2.44$, $SE = 0.09$) compared to tolerance ($M = 2.21$, $SE = 0.08$) were rated higher, irrespective of the environment shown [$b = 0.23$, $SE = 0.08$, $t_{(96)} = 2.90$, $p = .005$, $d_{rm} = 0.36$]. Post-hoc pairwise comparisons of the interaction effect revealed that comparing nature vs. urban [$b = 0.69$, $SE = 0.20$, $t_{(69.7)} = 3.40$, 95% CI = [0.135, 1.253], $p = .008$, $d_{rm} = 0.66$] and nature vs. indoor [$b = 1.04$, $SE = 0.19$, $t_{(75.2)} = 5.58$, $p < .001$, 95% CI = [0.530, 1.551], $d_{rm} = 1.06$] but not urban vs. indoor [$b = 0.35$, $SE = 0.17$, $t_{(82.2)} = 2.03$, 95% CI = [-0.120, 0.814], $p = .25$, $d_{rm} = 0.38$] was associated with higher ratings of distraction away from the painful episode. The same pattern was found for ratings of tolerance towards the painful stimulus, indicating significantly higher ratings of tolerance in nature when comparing nature vs. urban [$b = 1.10$, $SE = 0.20$, $t_{(69.7)} = 5.34$, $p < .001$, 95% CI = [0.543, 1.661], $d_{rm} = 1.03$] and nature vs. indoor [$b = 1.32$, $SE = 0.19$, $t_{(75.2)} = 7.12$, $p < .001$, 95% CI = [0.816, 1.837], $d_{rm} = 1.33$] but not urban vs. indoor [$b = 0.22$, $SE = 0.17$, $t_{(82.2)} = 1.32$, $p = .72$, 95% CI = [-0.243, 0.692], $d_{rm} = 0.25$]. The results mirror the findings observed using immediate self-reported pain by revealing effects specific to the nature condition. Compared to immediate ratings, they indicate effect sizes in the medium to high range when comparing nature to the other two conditions. Notably, comparisons between urban and indoor environments revealed small effect sizes, which did not reach statistical significance after correcting for multiple comparisons (Bonferroni-Holm).

Neural Responses to Pain. To establish whether our experimental design led to activity differences in regions and multivariate signature responses associated with pain processing, irrespective of (i.e., statistically orthogonal to) the conditions of interest, we performed a whole-brain, a region of interest (ROI), and a signature response analysis. For all analyses, a pain>no-pain contrast was created and thresholded using familywise-error (FWE) correction at voxel-level ($p < .05$). The contrast was calculated across all three environments, thus encompassing a total of 48 painful and 48 non-painful shocks. First, conducting the whole-brain analysis revealed extensive hemodynamic activity across several brain areas (Supplementary Table 3 and Supplementary Figure 1), including, among others, the anterior and posterior insula (aINS & pINS; bilateral), right primary somatosensory cortex (S1), secondary somatosensory cortex (S2; bilateral), anterior (ACC), and middle cingulate gyrus (MCC), superior frontal gyrus (SFG; including the supplementary motor area), cerebellum (bilateral), superior parietal lobe (SPL; bilateral), thalamus (bilateral) and periaqueductal grey (PAG). Second, we performed ROI analyses using preregistered sphere-based ROIs (Materials and Methods) extracted from previous meta-analyses on acute pain and studies using a similar pain paradigm conducted in our research group. These were the same ROIs as used in the main analyses of the manuscript, i.e., amygdala, anterior midcingulate cortex (aMCC), anterior (aINS) and posterior insula (pINS), medial prefrontal cortex (mPFC), primary (S1) and secondary somatosensory cortex (S2), periaqueductal grey (PAG),

superior parietal lobe (SPL), and thalamus. We extracted the percent signal change of each ROI using the MarsBar toolbox[16] for the pain>no-pain contrast and ran separate dependent t-tests comparing differences in signal change against zero (see Supplementary Table 4). The p-values for the ROI analyses were corrected by the overall number of investigated ROIs to account for multiple comparisons (p-values in Supplementary Table 4 represent adjusted values). As shown in Supplementary Table 4, all ROIs except the left (ipsilateral to the painful stimulation) and right primary somatosensory cortex ($p = .02$, one-sided, before multiple comparison correction) revealed a significant response. Third, we calculated the signature response for the NPS and SIIPS1 using the dot product of the contrast image (pain>no-pain) with the pattern map of the NPS or SIIPS1. Again, dependent t-tests were calculated comparing the signature response against zero, which revealed a significant result for the NPS ($t_{(48)} = 10.15$, $p < .001$) and SIIPS1 ($t_{(48)} = 5.96$, $p < .001$). Collectively, our analyses validate the efficacy of our pain paradigm in activating brain regions and multivariate signature responses typically associated with the first-hand experience of pain.

After establishing that our paradigm effectively led to neural signal changes associated with the first-hand experience of pain, we conducted our main analyses. The effects of interest are reported in the main manuscript. Here, we provide details regarding the remaining effects and detailed information of each LMM conducted.

First, we calculated a LMM using the signature responses (NPS and SIIPS1) as a dependent variable (Supplementary Table 5), to be predicted by the fixed effect of environment (nature as a reference), signature (NPS as reference), and their interaction (with random slopes and intercepts for environment and signature by participant). The signature response of the NPS and the SIIPS1 was standardized prior to calculating the model, resulting in a non-significant main effect of environment [$F_{(2,48.00)} = 2.29$, $p = .111$)] and signature [$F_{(1,48.00)} = 0.00$, $p = 1.00$)]; note that the second main effect cannot be meaningfully interpreted, as the responses were standardized in advance. However, since we were not interested in whether the overall response of both signatures, irrespective of the environment, would differ, we deliberately specified the model in this way. Importantly, and as mentioned in the main manuscript, there was a significant interaction effect between environment and signature [$F_{(2,96.00)} = 5.23$, $p = .006$)]. Planned pairwise comparisons revealed that there was a significant decrease in the NPS response during nature compared to the urban or indoor condition (see main manuscript). For the SIIPS1, no significant effects for the nature vs. urban [$\beta = 0.17$, $SE = 0.15$, $t_{(96.9)} = 1.14$, $p = .873$ one-tailed, 95% CI = [-0.126, 0.472], $d_{rm} = .18$] or indoor [$\beta = -0.23$, $SE = 0.18$, $t_{(79.3)} = -1.28$, $p = .101$ one-tailed, 95% CI = [-0.585, 0.126], $d_{rm} = -.22$] comparison were found, but a significant difference when comparing urban vs. indoor emerged [$\beta = -0.40$, $SE = 0.16$, $t_{(92.5)} = -2.58$, $p = .011$, 95% CI = [-0.712, -0. 093], $d_{rm} = -.40$]. The planned pairwise comparisons in the main manuscript were a priori not planned to be corrected for multiple comparisons, as they were based on assessing separate hypotheses. Notably, however, even when applying Bonferroni-Holm corrections for each dependent variable, the comparisons remained significant: nature vs. urban ($p = .018$) or indoor ($p = .020$) for the NPS, and urban vs. indoor ($p = .033$) for the SIIPS1.

As indicated above, we calculated a second LMM using a different operationalization of 'lower-level' vs. 'higher-level' pain components. For this, we specified a preregistered LMM in which, instead of the NPS and SIIPS1, all ROI responses related to sensory-discriminative (i.e., S1, S2, pINS, SPL) or affective-motivational (i.e., aMCC, aINS, mPFC, PAG) components were used as the dependent variable simultaneously. We included environment (nature as a reference), component (sensory-discriminative as a reference), and their interaction as fixed effects. Using this model (see details in Supplementary Table 6), we found significant results for the main effect of environment on the overall pooled ROI response [$F_{(2,48.00)} = 3.86$, $p = .028$], for the main effect of component [$F_{(1,48.00)} = 7.401$, $p = .009$], and importantly and as expected a significant interaction effect of environment*component [$F_{(2,96.00)} = 5.79$, $p = .004$]. Using planned pairwise comparisons revealed that there was a significant difference when comparing nature vs. urban [$b = -0.57$, $SE = 0.16$, $t_{(63.1)} = -3.52$, $p = .0004$, one-tailed, 95% CI = [-0.864, -0.239], $d_{rm} = -0.51$] and nature vs. indoor [$b = -0.57$, $SE = 0.19$, $t_{(57.0)} = -2.88$, $p = .0028$, 95% CI = [-0.963, -0.173], one-tailed, $d_{rm} = -0.44$] but not urban vs. indoor [$b = -0.02$, $SE = 0.18$, $t_{(58.5)} = -0.09$, $p = .93$, 95% CI = [-0.384, 0.350], $d_{rm} = -0.01$] when investigating the pooled activity of sensory-discriminative ROIs. For pooled activity of affective-motivational ROIs, no significant difference between nature vs. urban [$b = -0.18$, $SE = 0.16$, $t_{(63.1)} = -1.15$, $p = .13$ one-tailed, 95% CI = [-0.493, 0.132], $d_{rm} = -0.16$], nature vs. indoor

[b = -0.28, SE = 0.19, $t_{(57.0)}$ = -1.43, p = .079 one-tailed, 95% CI = [-0.677, 0.133], d_{rm} = -0.24] or urban vs. indoor emerged [b = -0.10, SE = 0.18, $t_{(58.5)}$ = -0.55, p = .58, 95% CI = [-0.469, 0.265], d_{rm} = -0.08]. Importantly, these results mirror the analysis presented in the main manuscript by showing that it is the 'lower-level' sensory-discriminative components rather than the 'higher-level' cognitive-emotional components that nature stimuli act on. Thus, using the initially planned analysis, we further corroborate the findings of the main manuscript by showing that nature also reduces pain when using a different indicator of 'lower-level' pain processing.

Second, we ran an individual LMM for each of the preregistered ROIs (Supplementary Figure 2). For ROIs with a sphere covering voxels in both hemispheres, we ran a LMM using the ROI response as the dependent variable to be predicted by the fixed effect of environment (nature as a reference, with random intercepts for participants). For ROIs with a separate sphere in each hemisphere, we used the ROI response of both hemispheres as a dependent variable to be predicted by the fixed effect of environment (nature as a reference), hemisphere (left as reference), and their interaction (with random slopes and intercepts for environment and hemisphere by participant).

For the main effect of environment we observed the following results for our ROIs: amygdala [$F_{(2,48.00)}$ = 2.06, p = .138)], aINS [$F_{(2,48.00)}$ = 3.08, p = .055)], aMCC [$F_{(2,96.00)}$ = 1.11, p = .335)], mPFC [$F_{(2,96.00)}$ = 0.101, p = .904)], PAG [$F_{(2,96.00)}$ = 0.31, p = .734)], pINS [$F_{(2,47.99)}$ = 18.62, p < .001)], S1 [$F_{(2,48.08)}$ = 2.37, p = .104)], S2 [$F_{(2,48.00)}$ = 7.84, p = .001)], SPL [$F_{(2,48.00)}$ = 3.98, p = .025)], thalamus [$F_{(2,48.00)}$ = 6.04, p = .005)]. Planned pairwise comparisons contrasting nature vs. urban and nature vs. indoor conditions for the ROIs with significant or trend level main effects of environment (i.e., pINS, S2, thalamus, aINS, SPL) are presented in the main manuscript and can be inspected in Supplementary Figure 3. Note that the reported p-values represent multiple comparison corrected values. Planned pairwise contrasts revealed no significant differences when comparing urban vs. indoor in these ROIs indicating that this effect was specific for comparisons involving the nature condition: thalamus [b = -0.12, SE = 0.10, $t_{(48)}$ = -1.17, p = .246, 95% CI = [-0.323, 0.085], d_{rm} = -0.16], S2 [b = -0.03, SE = 0.15, $t_{(48)}$ = -0.17, p = .864, 95% CI = [-0.328, 0.277], d_{rm} = -0.03], pINS [b = 0.41, SE = 0.20, $t_{(48)}$ = 2.05, p = .184, 95% CI = [0.007, 0.813], d_{rm} = -0.34], aINS [b = -0.19, SE = 0.17, $t_{(48)}$ = -1.18, p = .973, 95% CI = [-0.533, 0.138], d_{rm} = -0.17], and the SPL [b = -0.44, SE = 0.34, $t_{(48)}$ = -1.32, p = .582, 95% CI = [-1.110, 0.233], d_{rm} = -0.19]. For the ROIs with two spheres the main effect of hemisphere revealed the following results: amygdala [$F_{(1,48.00)}$ = 0.15, p = .698)], aINS [$F_{(1,48.00)}$ = 1.74, p = .192)], pINS [$F_{(1,48.00)}$ = 14.41, p < .001)], S1 [$F_{(1,63.55)}$ = 63.54, p < .001)], S2 [$F_{(1,47.99)}$ = 8.52, p = .005)], SPL [$F_{(1,48.00)}$ = 18.71, p < .001)], thalamus [$F_{(1,48.00)}$ = 3.34, p = .073)]. Regarding the same set of ROIs the interaction effect of environment*hemisphere revealed the following results: amygdala [$F_{(2,96.00)}$ = 3.63, p = .030)], aINS [$F_{(2,95.99)}$ = 0.43, p = .646)], pINS [$F_{(2,96.00)}$ = 0.68, p = .506)], S1 [$F_{(2,144.00)}$ = 1.02, p = .361)], S2 [$F_{(2,96.00)}$ = 4.80, p = .010)], SPL [$F_{(2,96.00)}$ = 0.14, p = .870)], thalamus [$F_{(2,96.00)}$ = 1.72, p = .183)]. Thus, concerning differences in the hemispheres, we found significant interactions of environment*hemisphere in the S2 and amygdala. Post-hoc tests indicated that differences in these ROIs were more pronounced in the left hemisphere (i.e., ipsilateral to the stimulated hand). All model details separated by ROI can be found in Supplementary Table 7–16.

Lastly, we conducted exploratory whole-brain analyses to explore putative activation differences outside the preregistered ROIs and signatures. We compared responses to painful>non-painful stimuli across environments (FWE-corrected at voxel level, p <.05). In the urban vs. nature comparison, we identified two clusters in the right (peak coordinate [64, -24, 8], cluster size = 588) and left superior temporal gyrus (peak coordinate [-56, -24, 10], cluster size = 426), localized in bilateral auditory cortex and thus suggesting differential effects of urban versus nature soundscapes on auditory processing. The indoor vs. nature comparison revealed a small cluster in the left thalamus (peak coordinate [-4, -18, 2], cluster size = 2), with voxels directly adjacent to those included in our predefined ROI for the thalamus. Beyond the differences in painful processing observed in our signature- and ROI-based confirmatory analyses, the exploratory whole-brain analyses indicate differences in auditory processing when comparing urban and nature environments (note that effects cannot be explained by differences in sound intensity, which had been normalized across urban and nature conditions).

Sensitivity analyses. We conducted several analyses investigating the sensitivity of our results to changes in analytical choices.

Exclusion of statistical outliers

We repeated all analyses reported in the main manuscript, excluding statistical outliers defined as values exceeding 3 standard deviations from the mean.

Applying this criterion led to the exclusion of two participants for the self-reported immediate pain ratings, four participants for the signature response data, and the following exclusions for the extracted ROIs: one participant for the Thalamus, two for S2, three for the pINS, two for the amygdala, one for the S1, six for the SPL, and three for the aINS.

Excluding these participants did not alter the main effect of *environment* [$F_{(2,46.12)} = 12.98$, $p < .001$] or the significant interaction between *environment*rating type* [$F_{(2,73.19)} = 8.51$, $p < .001$] for the immediate ratings. Similarly, the significant interaction effect between *environment*signature* [$F_{(2,88.91)} = 5.36$, $p = .006$] remained unchanged. For the ROIs we observed a significant main effect of *environment* in the Thalamus [$F_{(2,46.99)} = 7.45$, $p = .0015$], S2 [$F_{(2,45.99)} = 6.29$, $p = .0038$], and pINS [$F_{(2,44.99)} = 17.61$, $p < .001$]. However, the previously observed significant and trend-level effects for the SPL [$F_{(2,42.00)} = 1.86$, $p = .168$], and aINS [$F_{(2,45.00)} = 2.12$, $p = .133$] changed and were no longer significant.

Inspection of the planned pairwise comparisons for the significant main and interaction effects revealed similar overall results. Most significant planned pairwise comparisons remained significant after multiple comparison correction, with two exceptions. The comparison between nature and indoor environments shifted from significant to trend level in the NPS [b = -0.23, SE = 0.16, $t_{(82.3)} = -1.42$, $p = .079$ one-tailed, 95% CI = [-0.547, 0.091]] and the pINS [b = -0.41, SE = 0.19, $t_{(45)} = -2.09$, $p = .063$ one-tailed, 95% CI = [-0.795, -0.015]].

Exclusion of participants above motion threshold

We repeated all analyses reported in the main manuscript, excluding participants with head motion exceeding a threshold of 2 mm. This led to the exclusion of 9 participants, resulting in a final sample size of n = 40.

Excluding these participants did not affect the significant interaction between *environment*signature* [$F_{(2,116.99)} = 5.89$, $p = .004$] for the signature data. Regarding the ROIs, we observed a significant main effect of *environment* in the Thalamus [$F_{(2,39.00)} = 6.06$, $p = .005$], S2 [$F_{(2,39.00)} = 7.35$, $p = .0019$], and pINS [$F_{(2,39.00)} = 15.03$, $p < .001$]. However, the previously significant effect in the SPL shifted from significant to trend-level [$F_{(2,39.00)} = 3.04$, $p = .058$], and the previously observed trend for the aINS was no longer present [$F_{(2,39.00)} = 1.36$, $p = .266$].

Inspection of the planned pairwise comparisons for the significant main and interaction effects revealed similar overall results. Except for the following comparisons, all significant planned pairwise comparisons remained significant after multiple comparison corrections. The significant difference between nature and indoor environments in the pINS shifted to trend-level [b = -0.37, SE = 0.21, $t_{(39)} = -1.78$, $p = .053$ one-tailed, 95% CI = [-0.780, 0.049]]. Similarly, the previous trend-level effect in the SPL [b = -0.51, SE = 0.32, $t_{(39)} = -1.63$, $p = .11$ one-tailed, 95% CI = [-1.150, 0.125]] disappeared when comparing nature and urban environments.

Parsimonious first-level model

We repeated all analyses using an additional first-level model for the MRI data. Our original approach prioritized a parsimonious first-level model, which included 8 regressors for each environment: one regressor for delivery of painful shocks, one regressor for delivery of non-painful shocks, and six nuisance regressors accounting for motion. This approach was initially presented in the preprint of this article. After receiving feedback from the reviewers, re-examining the literature on similar research questions and designs[13,15], and principled arguments regarding the interpretability of the used implicit baseline, we reconsidered this approach and implemented an additional, more comprehensive first-level model. This model included 11 regressors for each environment: one regressor for the anticipation of painful shocks, one for the anticipation of non-painful shocks, one for delivery of painful shocks, one for delivery of non-painful shocks, one for the rating phase, and six nuisance regressors accounting for motion. The findings using this more comprehensive model, following validation checks orthogonal to the main hypotheses (see main

text), are now reported in the main text. For transparency and completeness, we also present the results of the more parsimonious first-level model here.

Using the more parsimonious model yielded the same significant interaction between *environment*signature* [$F_{(2,96.00)} = 6.04$, $p = .003$] for the signature data. Additionally, we observed the same significant main effects of environment in the Thalamus [$F_{(2,47.99)} = 5.53$, $p = .006$], S2 [$F_{(2,48.00)} = 5.16$, $p = .009$], and pINS [$F_{(2,48.00)} = 9.28$, $p = .0003$]. However, the previously significant and trend-level effects in the SPL [$F_{(2,48.00)} = 2.08$, $p = .136$] and aINS [$F_{(2,48.00)} = 2.39$, $p = .101$] were no longer observed.

Inspection of the planned pairwise comparisons for the significant main and interaction effects revealed the same overall results. There was no change in significance regarding the planned pairwise comparisons in the NPS, Thalamus, S2, and pINS.

Alternative ROIs

The main article reports ROIs that include only pain-sensitive voxels, identified using the pain>no-pain contrast across all environments. We focused on these pain-sensitive voxels due to the large size of the full ROI masks. Nevertheless, we also reran all analyses using the full ROIs, which included voxels both sensitive or insensitive to pain, and present these results here.

Using the full ROIs revealed the same significant main effect of environment in the Thalamus [$F_{(2,48.00)} = 5.94$, $p = .004$], S2 [$F_{(2,48.00)} = 7.73$, $p = .001$], and pINS [$F_{(2,48.00)} = 19.28$, $p < .001$]. However, the previously significant effect shifted to trend-level in the SPL [$F_{(2,48.00)} = 2.89$, $p = .064$], while the trend-level effect in the aINS became significant [$F_{(2,48.00)} = 3.30$, $p = .045$].

Inspection of the previously significant and trend-level planned pairwise comparisons revealed that the results remained consistent for the Thalamus, S2, pINS, aINS, and SPL after correcting for multiple comparisons.

Taken together, varying analytical approaches – such as excluding participants, altering the first-level design specifications, or using different ROI definitions – yielded consistent significant main and interaction effects for the signature data, Thalamus, S2, and pINS. Furthermore, the planned pairwise comparisons in these analyses remained highly consistent across different analytical choices. However, the main effect of environment and the planned pairwise comparisons in the SPL and aINS were more susceptible to these analytical variations, which indicates they should be interpreted with caution.

Additional preregistered analyses. As mentioned above, our preregistration included three additional hypotheses related to the impact of the environments on positive and negative affect and pulse rate, as well as the potential moderating role of nature connectedness on subjective and neural pain processing. Due to space constraints and because these hypotheses pertain to effects of nature that are only indirectly related to pain outcomes – placing them outside the core scope of this paper – these results are only briefly reported in the main text but fully documented here.

The effect of environments on positive and negative affect

Based on prior literature[17] we preregistered that nature exposure would lead to heightened positive and reduced negative affect compared to urban or indoor environments. Additionally, we expected these effects to be more pronounced for indicators of positive than for negative affect.

To address these hypotheses, participants completed the Positive and Negative Affect Schedule (PANAS) after each run in the experiment[18]. The PANAS measures subjective levels of positive and negative affect using 10 items each. Each item represents a specific feeling, and participants indicate on a scale from 1 (“very slightly or not at all”) to 5 (“extremely") how much the respective feeling applies to them at that moment. The two subscales of the PANAS – positive and negative affect – were calculated by averaging the items corresponding to each type of affect valence. To assess whether positive affect (PA) or negative affect (NA) differed across the three environments, we compared the PANAS ratings after each pain run, which included the presentation of painful and non-painful shocks alongside the environment. We calculated a LMM using affect (PA and NA) as the dependent variable, to be predicted by the fixed effect of environment (nature as a reference), valence (NA as a reference), and their interaction (with random slopes and intercepts for environment, valence, and their interaction by participant).

We observed a significant main effect of environment [$F_{(2,111.58)}$ = 9.09, p < .001)], valence [$F_{(2,48.00)}$ = 114.36, p < .001)], and their interaction [$F_{(2,49.02)}$ = 16.63, p < .001)]. In line with our predictions, planned pairwise comparisons revealed significantly lower negative affect in nature compared to urban [b = -0.33, SE = 0.06, $t_{(48)}$ = -5.18, p < .001 one-tailed, 95% CI = [-0.463, -0.204], d_{rm} = -0.76] or nature compared to indoor environments [b = -0.21, SE = 0.06, $t_{(48)}$ = -3.60, p < .001 one-tailed, 95% CI = [-0.322, -0.092], d_{rm} = -0.58]. Additionally, positive affect was significantly higher in nature compared to urban [b = 0.14, SE = 0.07, $t_{(48)}$ = 1.98, p = .026 one-tailed, 95% CI = [-0.002, 0.276], d_{rm} = 0.21] and nature compared to indoor environments [b = 0.34, SE = 0.07, $t_{(48)}$ = 4.78, p < .001 one-tailed, 95% CI = [0.195, 0.478], d_{rm} = 0.55]. We also found significantly higher positive affect [b = 0.20, SE = 0.07, $t_{(48)}$ = 2.80, p = .007, 95% CI = [0.056, 0.343], d_{rm} = 0.33], and higher negative affect [b = 0.13, SE = 0.06, $t_{(48)}$ = 2.02, p = .049, 95% CI = [0.001, 0.253], d_{rm} = 0.28] for urban compared to indoor environments.

Thus, in line with our prediction, receiving painful stimulation while being exposed to nature was associated with higher positive and lower negative affect compared to exposure to urban or indoor environments. Contrary to our prediction, the effect was more pronounced for negative (moderate to high effect size), than for positive affect (low to moderate effect size). Since our study, unlike most previous nature-based research, investigated affective processing in an aversive context (i.e., painful electrical stimulation), we speculate that this may have resulted in greater variability in negative affective responses, allowing for more pronounced differences across environments. Furthermore, we also observed differences when comparing urban and indoor environments, although these effects were generally smaller and less consistent in terms of their direction. Although the observed differences in self-reported affect are consistent with previous findings and appear to support stress recovery theory (SRT), the changes in neural pain outcomes do not suggest that the reduction in pain was driven by these affective changes (see discussion in main text).

The effect of environments on pulse rate

We preregistered that pulse rate will be decreased during exposure to nature as compared to urban environments. Since we were not aware of studies comparing differences in pulse rate for nature and indoor environments, we restricted the predictions to the nature vs. urban comparison.

To investigate this hypothesis, we recorded pulse rate using the built-in peripheral pulse unit of the MRI scanner (PPU, Siemens Medical, Erlangen, Germany). The raw photoplethysmography (PPG) signal, sampled at 200 Hz, was processed for each functional run. Pulse rate was derived from the intervals between pulse peaks and is expressed in beats per minute (BPM). Signal processing and RR interval detection were performed using the Python Heart Rate Analysis Toolkit heartpy[19]. Each participant's data were manually inspected to ensure accurate peak detection and to identify any missed or incorrectly detected peaks. One participant was excluded from the analysis due to an erroneous raw PPG signal, which could not be processed. We calculated a LMM using BPM as the dependent variable, to be predicted by the fixed effect of environment (nature as a reference, with random intercepts for environment by participant).

We observed no significant main effect of environment. However, since we were interested in the difference between nature and urban environments, we continued to perform a planned pairwise comparison. This comparison revealed significantly lower BPM for the nature environment when compared to the urban environment, with a small effect size [b = -0.86, SE = 0.42, $t_{(94)}$ = -2.04, p = .043, 95% CI = [-1.700, -0.025], d_{rm} = -0.09]. Exploratory post-hoc pairwise comparisons revealed no difference when comparing the nature and the indoor environment [b = -0.42, SE = 0.42, $t_{(94)}$ = -0.99, p = .321, 95% CI = [-1.260, 0.417], d_{rm} = -0.05] or the urban and indoor environment [b = 0.44, SE = 0.42, $t_{(94)}$ = 1.05, p = .298, 95% CI = [-0.397, 1.280], d_{rm} = 0.05].

Thus, in line with our prediction, pulse rate was significantly lower when comparing nature and urban environments. However, the effect size was very small and translated to a change of approximately one BPM between nature (M = 65.09, SD = 9.05) and urban environments (M = 65.96, SD = 9.44). Moreover, the lack of differences to the neutral indoor environment makes it difficult to interpret the underlying mechanism of these differences.

The effect of nature connectedness on the impact of environment

Drawing on existing literature that demonstrated the beneficial effects of nature are influenced by individual levels of nature connectedness[20], we preregistered our hypothesis that participants with a stronger connection to nature will exhibit greater changes in pain responses when exposed to nature.

To investigate our hypothesis, we used the Nature Connection Index (NCI) to assess individual levels of nature connectedness[21]. The NCI comprises six items (e.g., “I always find beauty in nature”) rated on a 7-point scale, ranging from “completely disagree” to “completely agree”. Following the authors’ recommendations, we calculated overall levels of nature connectedness by using a weighted point index, where higher scores indicate greater nature connectedness. In the subsequent analyses, we recalculated all models that included significant main or interaction effects of the variable environment on immediate self-reported or neural data. We included nature connectedness (centered) and its interaction with the other main effects as independent variables. For the ROI data, we focused the analyses on the thalamus, S2 and pINS, as these regions were characterized by robust effects of environment across different analytical choices (see Sensitivity analyses above).

For the immediate pain ratings, we employed a LMM with ratings as the dependent variable, to be predicted by the fixed effect of environment (nature as a reference), rating content (intensity as a reference), nature connectedness and their interactions (with random intercepts and slopes for environment, rating content, and their interactions). For the signature data, we recalculated a LMM using signature responses as the dependent variable, to be predicted by the effect of environment (nature as a reference), signature (NPS as a reference), nature connectedness and their interactions (with random intercepts and slopes for environment). Notably, we encountered convergence issues when including the random effect of signature in the moderator model. Consequently, this random effect was excluded from both models in the moderator analysis. Importantly, this exclusion did not affect the overall interpretation of the model. Furthermore, for the thalamus, S2, and pINS, we recalculated a LMM using the ROI response as the dependent variable, to be predicted by the fixed effect of environment (nature as a reference), hemisphere (left hemisphere as a reference), nature connectedness and their interactions (with random intercepts and slopes for environment and hemisphere). We calculated likelihood ratio tests (LRT) to compare the performance of two models per dependent variable: one model including nature connectedness as a moderator variable and one excluding it. A significant result from the LRT indicates that the more complex model, which incorporates nature connectedness as a fixed effect, provides a significantly better fit to the data compared to the simpler model. Additionally, we checked the models for significant main effects of nature connectedness or its interaction with the variable environment.

The results of the LRTs are summarized in Supplementary Table 17. We did not observe significant improvements in model fit when incorporating nature connectedness as a fixed effect into our models. Furthermore, neither a significant main effect of nature connectedness nor any significant interactions with the variable environment were detected. Note, that the LRT for the thalamus responses approached trend-level significance. However, the main effect of nature connectedness, as well as its two-way and three-way interactions with environment, did not reach significance (all $p > .34$).

Thus, contrary to our preregistered hypothesis, our data did not reveal that nature connectedness significantly altered the impact of nature on self-reported and neural responses to pain. This contrasts with the literature suggesting that the beneficial effects of nature are moderated by nature connectedness[20], a discrepancy that may arise from differences in outcome variables or the type of nature exposure investigated. Previous studies demonstrating this moderation effect focused on measures of mental health and well-being and examined the effect of real-world nature exposure. It is possible that the effects on mental health and well-being, which are less tangible than immediate pain experiences in response to a specific stimulus, may be more pronounced. Furthermore, virtual nature may not provide the same increased benefits for individuals with higher levels of nature connectedness, as it could differ significantly from their typical experiences in nature. However, employing non-real, virtual nature rather than real-world stimuli also minimized potential confounding influences stemming from familiarity and prior experiences with specific settings. Notably, such prior experiences and learned associations may affect the salutary effects of nature. It has been suggested that positive past interactions can shape the restorative effects of

nature through conditioning processes[22]. When positive emotions are repeatedly linked with specific environments, cues from these environments may eventually elicit similar positive responses. Additionally, positive past experiences are related to high levels of nature connectedness which in turn are associated with greater perceived benefits from nature[23]. Since we found no moderating effect of nature connectedness – an outcome typically associated with positive prior experiences - on the pain outcomes, our current findings do not appear to support these suggestions. However, these interpretations are speculative, especially as nature connectedness is only an indirect measure of positive past experiences. Thus, future studies should investigate more directly the influence of prior experiences and learning mechanisms on the pain-relieving effects of nature.

Additional non-preregistered analyses. In addition to the preregistered hypotheses and the sensitivity analyses reported above, we performed exploratory analyses to further investigate associations between self-reported and neural pain responses. These analyses aimed to explore potential explanations for our primary findings.

First, we conducted correlation analyses to examine the relationship between self-reported and neural responses to pain. Specifically, we aggregated immediate pain ratings – separated by intensity and unpleasantness – and neural responses for the NPS, SIIPS1, Thalamus, S2, and pINS, separately across environments. Our analyses focused on neural responses that demonstrated robust, significant differences between environments as reported in the Sensitivity Analyses section. The correlation analyses revealed moderate positive associations between neural responses and self-reported pain intensity with correlations of r = .32 (p = .024) for the NPS and r. = .40 (p = .009) for the SIIPS1 response. Similarly, these responses were moderately associated with unpleasantness ratings (r = .49, p = .001; and r = .50, p = .001; respectively). All associations between signature responses and ratings were significant after multiple comparison corrections across the four correlations (Bonferroni-Holm; reported p-values represent adjusted values). We also observed weak positive, but non-significant correlations between the thalamus (r = .09, p = 1), S2 (r = .20, p = .51), and pINS (r = .05, p = 1) responses and pain intensity. Notably, these regions showed slightly stronger correlations with unpleasantness ratings (r = .28, p = .25; r = .33, p = .12; and r = .24, p = .36; respectively). Applying multiple comparison correction across the six correlations (Bonferroni-Holm; reported p-values represent adjusted values) rendered the correlations between ROI responses and unpleasantness ratings as not significant anymore. Thus, higher self-reported pain was significantly associated with higher neural responses in the signature but not the ROI data. The magnitude of these associations is consistent with prior studies, which report weak to moderate associations between self-reported pain and neural responses to pain[14,24]. Furthermore, in an exploratory additional analysis prompted by a reviewer comment, we calculated difference scores for immediate self-reported pain ratings and neural responses (NPS, SIIPS1, Thalamus, and pINS) between pairs of environments. Specifically, we computed the difference between the average response in the natural environment and the average response in the urban or indoor environments, separately for each dependent variable. Additionally, we calculated the difference between the average response in the urban and indoor environments. We then explored the correlations of these difference scores between self-reported pain ratings and neural responses for each environment pair (e.g., the correlation between the nature-urban difference in intensity ratings and Thalamus activity). For the nature-urban difference, we observed the following correlations: r = .19 (p = .19), r = .23 (p = .11), r = .38 (p = .007), r = .29 (p = .043), and r = .19 (p = .183) for intensity ratings, and r = .27 (p = .064), r = .20 (p = .168), r = .43 (p = .002), r = .30 (p = .034), and r = .33 (p = .021) for the unpleasantness ratings with the NPS, SIIPS1, Thalamus, S2, and pINS responses, respectively. For the nature-indoor difference, we observed similar correlations: r = .19 (p = .19), r = .24 (p = .11), r = .15 (p = .319), r = .23 (p = .110), and r = .18 (p = .207) for intensity ratings, and r = .25 (p = .085), r = .19 (p = .182), r = .26 (p = .071), r = .40 (p = .004), and r = .33 (p = .018) for the unpleasantness ratings with the NPS, SIIPS1, Thalamus, S2, and pINS responses, respectively. Finally, for the urban-indoor difference, we observed the following correlations: r = .03 (p = .843), r = .04 (p = .805), r = .01 (p = .930), r = -.09 (p = .559), and r = -.13 (p = .371) for intensity ratings, and r = .14 (p = .336), r = -.11 (p = .452), r = .12 (p = .395), r = .13 (p = .386), and r = .13 (p = .361) for the unpleasantness ratings with the NPS, SIIPS1, Thalamus, S2, and pINS responses, respectively. Note that the correlations were not corrected for

multiple comparisons due to the exploratory nature of this analysis. In general, we observed that larger differences in neural responses were associated with larger differences in self-reported ratings, with the most robust associations found for differences in nociception-related areas (i.e., Thalamus, S2). Notably, this was not substantiated by the associations observed between differences in self-reported ratings and the NPS or SIIPS1, which exhibited similarly modest correlations. Furthermore, the overall pattern revealed relatively low correlations, which is not surprising, given the inherent complexity of the measures and the well-documented finding that self-reported and neural pain data typically exhibit low to moderate associations, which was also confirmed by our study (see correlations reported above). Since these correlations accounted for 25% or less of the variance in neural responses (or vice versa) across environments in our data, the potential magnitude of the exploratory correlations of the differences across environments is inherently constrained. Thus, these findings highlight the value of integrating both neural and self-reported pain measures, as they offer complementary insights into pain processing. While self-reported pain reflects the subjective experience, neural data can reveal the underlying brain mechanisms involved in pain perception[25,26].

Second, we examined whether the three environments differed in terms of immersion. Following previous research[27] we assessed subjective immersion using three items: Presence ("I felt present in the environment shown"), involvement ("I have forgotten the real environment around me"), and realism ("The environment shown felt very realistic"). Each item was rated on a scale from 1 ("not at all") to 5 ("very"). We calculated the average score of the three items (Cronbach's $\alpha = .81$) to estimate the overall immersion for each environment. To test for differences in immersion, we ran a LMM with the average immersion score as the dependent variable and environment as the predictor (nature as a reference, with random intercepts per participant). We found a significant effect of environment [$F_{(2,96)} = 16.62$, $p < .001$)]. Post-hoc pairwise comparisons revealed that the nature environment was significantly more immersive compared to the urban [$b = 0.28$, $SE = 0.11$, $t_{(96)} = 2.54$, $p = .034$, 95% CI = [0.017, 0.541], $d_{rm} = .36$], or the indoor environments [$b = 0.63$, $SE = 0.11$, $t_{(96)} = 5.75$, $p < .001$, 95% CI = [0.371, 0.894], $d_{rm} = .80$]. Additionally, the urban environment was rated as significantly more immersive than the indoor environment [$b = 0.35$, $SE = 0.11$, $t_{(96)} = 3.22$, $p = .005$, 95% CI = [0.092, 0.616], $d_{rm} = .47$].

Third, to explore whether immersion levels explained differences in neural and self-reported responses to pain, we ran several additional LMMs. For these LMMs we used immersion (centered) as a covariate in all robust pain responses identified in our main analyses (see Sensitivity Analysis above). For immediate self-reported pain, we conducted a LMM with pain ratings as the dependent variable, to be predicted by the fixed effect of immersion, environment (nature as a reference), rating content (intensity as a reference), and the interaction of environment*rating content (with random intercepts and slopes for environment, rating content, and their interaction). For the signature responses, we performed a similar LMM with signature responses as the dependent variable, to be predicted by the effect of immersion, environment (nature as a reference), signature (NPS as a reference), and the environment*signature interaction (with random intercepts and slopes for environment and signature). Additionally, for the Thalamus, S2, and pINS, we ran LMMs using the ROI response as the dependent variable, to be predicted by the fixed effect of immersion, environment (nature as a reference), hemisphere (left hemisphere as a reference), and the environment*hemisphere interaction (with random intercepts and slopes for environment and hemisphere).

The results of the analyses revealed no significant main effect of immersion for the immediate ratings, signature, S2 and pINS models (all $p > .19$). For the Thalamus, immersion showed a trend-level result of $p = .067$. However, for all models the previously identified significant main and interaction effects of environment stayed significant.

Thus, although the three environments showed differences in their absolute levels of self-reported immersion, we found no indication that immersion levels contributed significantly to the observed differences in self-reported and neural responses to pain. Additionally, we found the same main and interaction effects of environment on pain after controlling for levels of immersion. These findings support the conclusion that immersion likely did not drive the observed effects.

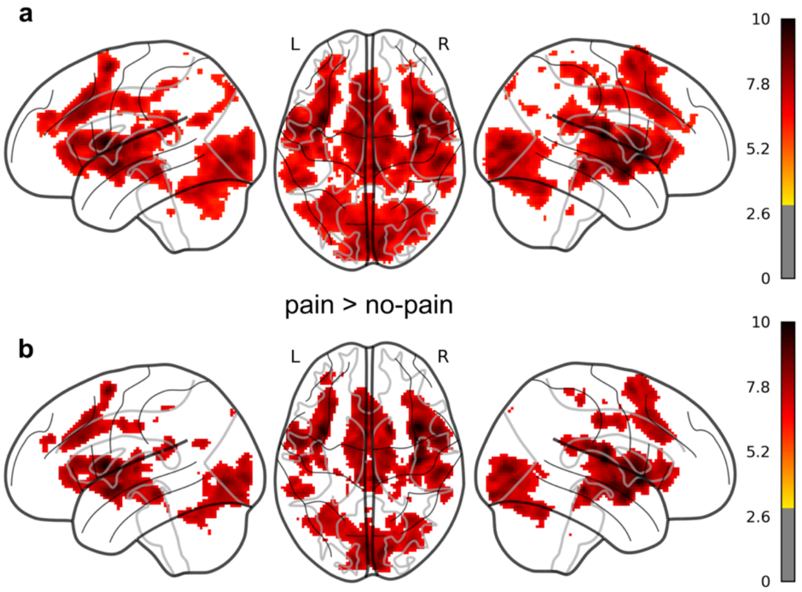

Supplementary Fig. 1 Whole-brain results of pain>no-pain contrast across environments. Since the identified clusters at the a priori FWE-corrected threshold turned out to be rather large, we present the statistical activation maps at two thresholds to facilitate the identification and delineation of activated regions: **a** A priori threshold with FWE-correction at $p < .05$; **b** More conservative threshold with FWE-correction at $p < .001$. The glass brain shows activity changes in the hemodynamic response triggered by electrical stimulations of the left hand. We observed increased activity in a wide variety of brain regions associated with processing first-hand pain. L = left hemisphere, R = right hemisphere; color bar depicts t-values. Source data are provided as a Source Data file.

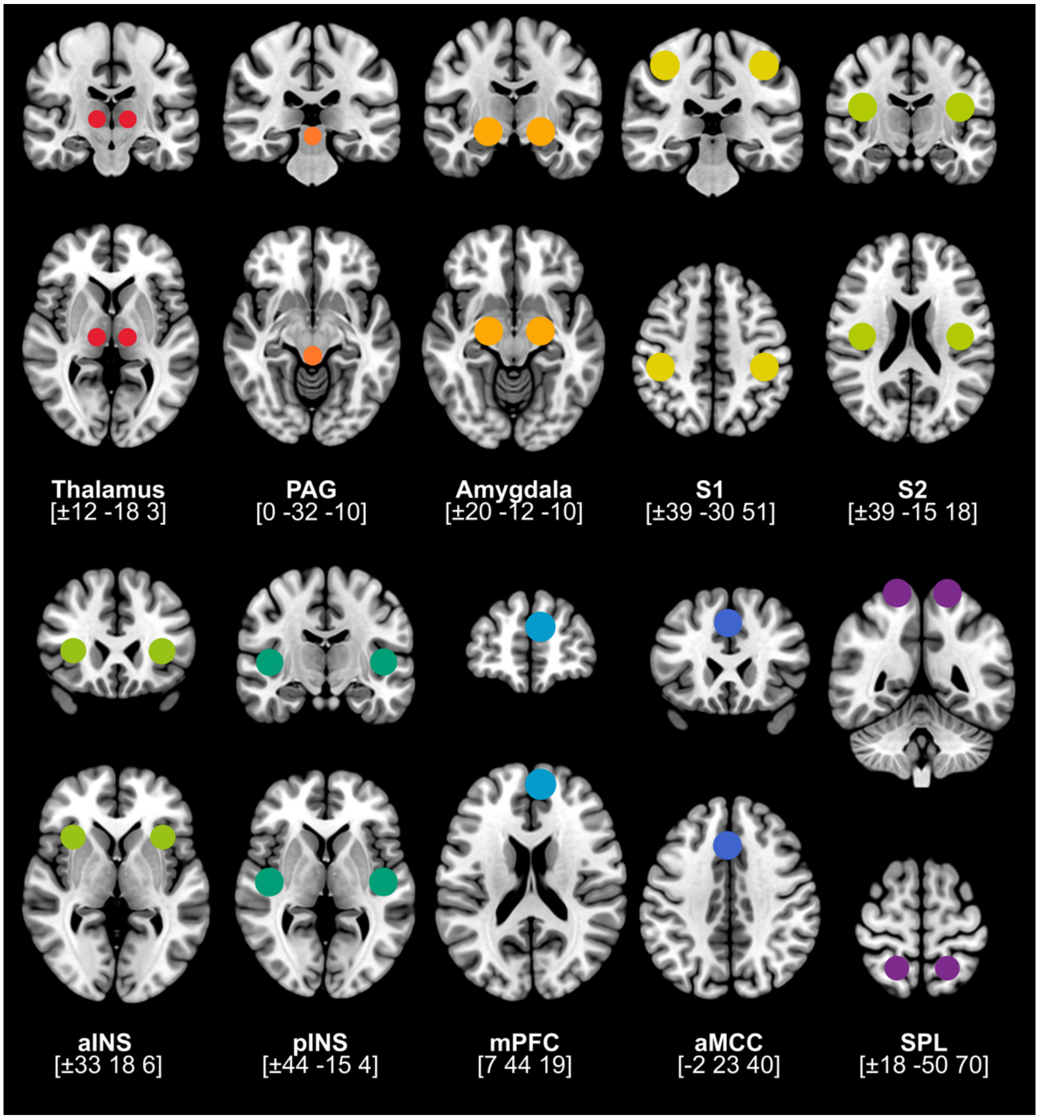

Supplementary Fig. 2 Regions of interest for comparing neural pain responses. The selection of regions of interest (ROIs; center [± x, y, z]; sphere size) was guided by a theory-based approach, focusing on regions involved in three key neural circuits as outlined in the framework proposed by Bushnell et al.[28]. The first circuit represents the ascending pathway, which includes the left/right primary somatosensory cortex (S1; [±39 -30 51], 10mm) and the left/right thalamus ([±12, -18, 3]; 6mm). The second circuit is linked to attentional modulations of pain. It involves the left/right superior parietal lobe (SPL; [±18, -50, 70]; 10mm), the left/right secondary somatosensory cortex (S2; [±39, -15, 18]; 10mm), the left/right posterior insula (pINS; [±44, -15, 4]; 10mm), and the left/right amygdala ([±20, -12, -10]; 10mm). The third circuit is associated with emotional modulation of pain, encompassing the left/right anterior insula (aINS; [±33, 18, 6]; 10mm), the anterior midcingulate cortex (aMCC; [-2, 23, 40], 10mm), the medial prefrontal cortex (mPFC; [7, 44, 19]; 10mm), and the periaqueductal gray (PAG; [0, -32, -10]; 6mm).

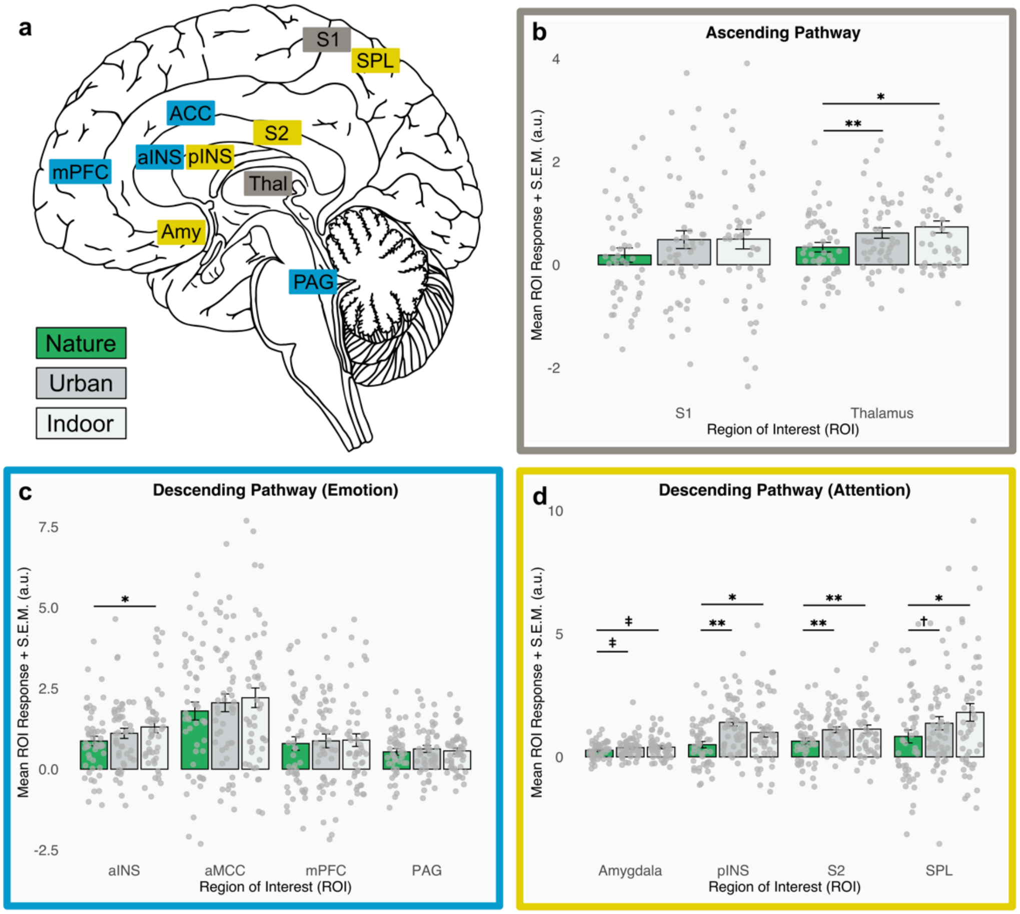

Supplementary Fig. 3 Activity differences in regions of interest across environments. Regions of interest (ROI) are organized in three key neural circuits as outlined in the framework proposed by Bushnell et al.[28]. **a** Schematic representation of neural circuits and associated ROIs. Grey, blue, and yellow ROIs belong to the ascending, descending (emotion), and descending (attention) pain pathways respectively (adapted with permission from Bushnell et al.[28]). **b** Bar-plot of mean contrast estimates and standard errors within the primary somatosensory cortex (S1) and the Thalamus per environment. **c** Bar-plot of mean contrast estimates and standard errors within the anterior insula (aINS), anterior midcingulate cortex (aMCC), medial prefrontal cortex (mPFC), and periaqueductal grey (PAG) per environment. **d** Bar-plot of mean contrast estimates and standard errors within the amygdala, posterior insula (pINS), secondary somatosensory cortex (S2) and superior parietal lobe (SPL). The three plots depict individual ROI responses (grey dots) for each environment and participant (n = 49) separated by ROIs. The bars represent estimated marginal means with standard errors of the mean (S.E.M.) derived from the linear mixed models. † < .1, * < .05, ** < .01, mark trend-level and significant planned pairwise comparisons; ‡ marks trend-level planned pairwise comparisons in absence of a significant main effect of environment in the linear mixed model. Planned pairwise comparisons (one-sided, Bonferroni-Holm corrected for each pathway) are derived from the mixed effects models. a.u. = arbitrary units. Source data are provided as a Source Data file.

Supplementary Table 1. Linear mixed model results for immediate subjective pain ratings (i.e., intensity and unpleasantness).

Fixed Effects

	Estimate	SE	95% CI	t	p
Intercept	5.47	0.14	5.202 – 5.736	40.38	<.000001
Nat vs. Urb	0.25	0.12	0.019 – 0.476	2.14	.037
Nat vs. Ind	0.29	0.11	0.077 – 0.509	2.67	.009
Int vs. Unp	-0.81	0.14	-1.081 – -0.538	-5.87	<.000001
Nat vs. Urb * Int vs. Unp	0.58	0.14	0.311 – 0.851	4.23	.00009
Nat vs. Ind * Int vs. Unp	0.36	0.13	0.109 – 0.623	2.80	.006

Random Effects

	Variance	SD	Correlation				
Participant (Intercept)	0.732	0.855					
Nat vs. Urb (Slope)	0.326	0.571	-.33				
Nat vs. Ind (Slope)	0.255	0.505	-.24	.39			
Int vs. Unp (Slope)	0.597	0.773	.20	-.45	-.16		
Nat vs. Urb (Slope) * Int vs. Unp	0.257	0.507	-.31	.55	.27	-.28	
Nat vs. Ind (Slope) * Int vs. Unp	0.169	0.411	-.16	.59	.89	-.14	.62

Model fit

R^2	Marginal	Conditional
	0.12	0.55

Note: p-values for fixed effects calculated using Satterthwaites approximations. All p-values are based on two-sided tests (uncorrected). Confidence Intervals (CI) have been calculated using the Wald method. SE = standard error, SD = standard deviation. Model equation: Immediate_Pain ~ Environment*Content + (1+Environment*Content|Participant). Nat = Nature, Urb = Urban, Ind = Indoor, Int = Intensity, Unp = Unpleasant. Source data are provided as a Source Data file.

Supplementary Table 2. Linear mixed model results for retrospective subjective pain ratings (i.e., distraction and tolerance).

Fixed Effects					
	Estimate	SE	95% CI	t	p
Intercept	3.02	0.57	1.90 – 4.14	5.31	<.000001
Nat vs. Urb	0.94	0.80	-0.64 – 2.52	1.17	.243
Nat vs. Ind	0.10	0.79	-1.36 – 1.57	0.13	.898
Dis vs. Tol	~0.00	0.12	-0.24 – 0.24	0.00	1.000
Nat vs. Urb * Dis vs. Tol	-0.41	0.17	-0.73 – -0.09	-2.36	.019
Nat vs. Ind * Dis vs. Tol	-0.29	0.17	-0.60 – 0.03	-1.65	.101

Random Effects				
	Variance	SD	Correlation	
Participant (Intercept)	0.816	0.903		
Nat vs. Urb (Slope)	1.351	1.162	-.72	
Nat vs. Ind (Slope)	1.014	1.007	-.80	.70

Model fit		
R^2	Marginal	Conditional
	0.22	0.71

Note: p-values for fixed effects calculated using Satterthwaites approximations. All p-values are based on two-sided tests (uncorrected). Confidence Intervals (CI) have been calculated using the Wald method. SE = standard error, SD = standard deviation. Model equation: Retrospective_Pain ~ Environment*Content + (1+Environment|Participant). Nat = Nature, Urb = Urban, Ind = Indoor, Dis = Distraction, Tol = Tolerance. Source data are provided as a Source Data file.

Supplementary Table 3. Whole brain results for the contrast pain>no-pain across all environments.

Clusters and brain regions	h	MNI			t-value	p
		x	y	z		
Cluster 1 (k = 31099)	R	38	10	-2	10.43	<.000001
Insular Cortex (anterior)	L	-34	8	10	10.16	
Insular Cortex (posterior)	R	38	-16	12	10.13	
Cluster 2 (k = 13893)	R	12	-84	6	9.19	<.000001
Lingual Gyrus	L	-2	-80	-2	8.95	
Occipital Pole	R	8	-96	-14	8.92	

Note: Significant clusters resulting from the contrast [pain>no-pain]. h = hemisphere, *k* = cluster size, MNI coordinates x, y, z, *t*-value, and *p*-value (FWE-corrected at $p < .05$; cluster-level for Cluster 1 and 2; voxel-level for sub-clusters). Peak coordinates were labeled according to the automated anatomical labeling (AAL2) atlas[29].

Supplementary Table 4. Region of interest results for the contrast pain>no-pain.

**Region**	df	M	95% CI	*t*-value	*p*
Amygdala (L)	48	0.24	0.140 – 0.335	4.90	.0002
Amygdala (R)	48	0.19	0.096 – 0.281	4.12	.0023
Anterior insula (L)	48	0.96	0.727 – 1.210	8.08	< .000001
Anterior insula (R)	48	0.94	0.698 – 1.183	7.80	< .000001
Anterior midcingulate cortex	48	1.96	1.500 – 2.428	8.52	< .000001
Medial prefrontal cortex	48	0.46	0.212 – 0.707	3.73	.008
Periaqueductal grey	48	0.55	0.403 – 0.704	7.31	< .000001
Posterior insula (L)	48	0.80	0.597 – 1.003	7.93	< .000001
Posterior insula (R)	48	0.99	0.736 – 1.239	7.91	< .000001
Primary somatosensory cortex (L)	48	0.02	-0.236 – 0.284	0.18	.909
Primary somatosensory cortex (R)	48	0.29	0.001 – 0.581	2.00	.264
Secondary somatosensory cortex (L)	48	0.71	0.501 – 0.910	6.94	< .000001
Secondary somatosensory cortex (R)	48	1.01	0.796 – 1.227	9.41	< .000001
Superior parietal lobe (L)	48	0.69	0.349 – 1.039	4.04	.003
Superior parietal lobe (R)	48	1.16	0.723 – 1.593	5.35	.00003
Thalamus (L)	48	0.49	0.338 – 0.646	6.43	< .000001
Thalamus (R)	48	0.58	0.405 – 0.750	6.74	< .000001

Note: All p-values are based on t-tests against 0 and represent adjusted values (Bonferroni corrected; two-sided); M = Mean; CI = Confidence Interval. Source data are provided as a Source Data file.

Supplementary Table 5. Linear mixed model results for multivariate signature responses (i.e., NPS and SIIPS1).

Fixed Effects					
	Estimate	SE	95% CI	t	p
Intercept	-0.27	0.13	-0.52 – -0.01	-2.08	.041
Nat vs. Urb	0.38	0.15	0.05 – 0.69	2.55	.012
Nat vs. Ind	0.42	0.18	-0.05 – 0.66	2.59	.021
NPS vs. SIIPS1	0.25	0.13	-0.03 – 0.53	1.86	.065
Nat vs. Urb * NPS vs. SIIPS1	-0.55	0.18	-0.97 – -0.25	-3.18	.002
Nat vs. Ind * NPS vs. SIIPS1	-0.19	0.18	-0.49 – 0.23	-1.09	.277

Random Effects					
	Variance	SD	Correlation		
Participant (Intercept)	0.438	0.662			
Nat vs. Urb (Slope)	0.361	0.600	-.61		
Nat vs. Ind (Slope)	0.808	0.899	-.14	.67	
NPS vs. SIIPS1 (Slope)	0.129	0.359	-.20	-.01	-.19

Model fit		
R^2	Marginal	Conditional
	0.03	0.63

Note: p-values for fixed effects calculated using Satterthwaites approximations. All p-values are based on two-sided tests (uncorrected). Confidence Intervals (CI) have been calculated using the Wald method. SE = standard error, SD = standard deviation. Model equation: Signature_Response ~ Environment*Signature + (1+Environment+Signature|Participant). Nat = Nature, Urb = Urban, Ind = Indoor, NPS = neurologic pain signature, SIIPS1 = stimulus intensity independent pain signature-1. Source data are provided as a Source Data file.

Supplementary Table 6. Linear mixed model results for pooled region of interest (ROI) responses (i.e., ROIs clustered into sensory-discriminative and affective-motivational components).

Fixed Effects					
	Estimate	SE	95% CI	t	p
Intercept	0.97	0.15	0.67 – 1.28	6.31	<.00001
Nat vs. Urb	0.18	0.16	-0.13 – 0.49	1.15	.253
Nat vs. Ind	0.28	0.19	-0.11 – 0.67	1.43	.157
Aff-Mot vs. Sen-Dis	-0.44	0.10	-0.64 – -0.23	-4.21	.0001
Nat vs. Urb * Aff-Mot vs. Sen-Dis	0.37	0.11	0.15 – 0.60	3.25	.002
Nat vs. Ind * Aff-Mot vs. Sen-Dis	0.28	0.11	0.06 – 0.51	2.50	.013

Random Effects					
	Variance	SD	Correlation		
Participant (Intercept)	1.009	1.004			
Nat vs. Urb (Slope)	0.880	0.938	-.35		
Nat vs. Ind (Slope)	1.585	1.259	-.42	.48	
Aff-Mot vs. Sen-Dis (Slope)	0.203	0.451	-.31	.05	.24

Model fit		
R^2	Marginal	Conditional
	0.04	0.89

Note: p-values for fixed effects calculated using Satterthwaites approximations. All p-values are based on two-sided tests (uncorrected). Confidence Intervals (CI) have been calculated using the Wald method. SE = standard error, SD = standard deviation. Model equation: Pooled_ROI_Response ~ Environment*Component + (1+Environment+Component|Participant). Nat = Nature, Urb = Urban, Ind = Indoor, Aff-Mot = affective-motivational, Sen-Dis = sensory-discriminative. Source data are provided as a Source Data file.

Supplementary Table 7. Linear mixed model results for the amygdala response.

Fixed Effects					
	Estimate	SE	95% CI	t	p
Intercept	0.25	0.05	0.14 – 0.35	4.47	.00004
Nat vs. Urb	0.16	0.07	0.03 – 0.29	2.45	.017
Nat vs. Ind	0.13	0.07	-0.01 – 0.27	1.80	.077
Left vs. Right	0.05	0.03	-0.02 – 0.12	1.31	.194
Nat vs. Urb * Left vs. Right	-0.11	0.05	-0.20 – -0.02	-2.40	.018
Nat vs. Ind * Left vs. Right	0.01	0.05	-0.10 – 0.08	-0.13	.892

Random Effects					
	Variance	SD	Correlation		
Participant (Intercept)	0.122	0.350			
Nat vs. Urb (Slope)	0.161	0.401	-.37		
Nat vs. Ind (Slope)	0.203	0.451	-.39	.58	
Left vs. Right (Slope)	0.017	0.131	-.45	.14	.22

Model fit		
R^2	Marginal	Conditional
	0.02	0.87

Note: p-values for fixed effects calculated using Satterthwaites approximations. All p-values are based on two-sided tests (uncorrected). Confidence Intervals (CI) have been calculated using the Wald method. SE = standard error, SD = standard deviation. Model equation: Amy_Response ~ Environment*Hemisphere + (1+Environment+Hemisphere|Participant). Nat = Nature, Urb = Urban, Ind = Indoor. Source data are provided as a Source Data file.

Supplementary Table 8. Linear mixed model results for the anterior insula (aINS) response.

Fixed Effects					
	Estimate	SE	95% CI	t	p
Intercept	0.83	0.15	0.54 – 1.13	5.62	<.000001
Nat vs. Urb	0.22	0.16	-0.09 – 0.54	1.42	.162
Nat vs. Ind	0.45	0.18	0.10 – 0.81	2.53	.015
Left vs. Right	0.07	0.06	-0.06 – 0.20	1.10	.272
Nat vs. Urb * Left vs. Right	0.02	0.08	-0.12 – 0.17	0.33	.743
Nat vs. Ind * Left vs. Right	-0.04	0.08	-0.19 – 0.10	-0.59	.554

Random Effects					
	Variance	SD	Correlation		
Participant (Intercept)	1.011	1.005			
Nat vs. Urb (Slope)	1.088	1.042	-.45		
Nat vs. Ind (Slope)	1.461	1.208	-.40	.50	
Left vs. Right (Slope)	0.079	0.282	-.06	.06	.13

Model fit		
R^2	Marginal	Conditional
	0.02	0.95

Note: p-values for fixed effects calculated using Satterthwaites approximations. All p-values are based on two-sided tests (uncorrected). Confidence Intervals (CI) have been calculated using the Wald method. SE = standard error, SD = standard deviation. Model equation: aINS_Response ~ Environment*Hemisphere + (1+Environment+Hemisphere|Participant). Nat = Nature, Urb = Urban, Ind = Indoor. Source data are provided as a Source Data file.

Supplementary Table 9. Linear mixed model results for the anterior midcingulate cortex (aMCC) response.

Fixed Effects					
	Estimate	SE	95% CI	t	p
Intercept	1.79	0.29	1.23 – 2.36	6.29	<.000001
Nat vs. Urb	0.25	0.28	-0.30 – 0.80	0.91	.363
Nat vs. Ind	0.41	0.28	-0.14 – 0.96	1.47	.144

Random Effects		
	Variance	SD
Participant (Intercept)	2.10	1.44

Model fit		
R^2	Marginal	Conditional
	0.007	0.53

Note: p-values for fixed effects calculated using Satterthwaites approximations. All p-values are based on two-sided tests (uncorrected). Confidence Intervals (CI) have been calculated using the Wald method. SE = standard error, SD = standard deviation. Model equation: aMCC_Response ~ Environment + (1|Participant). Nat = Nature, Urb = Urban, Ind = Indoor. Source data are provided as a Source Data file.

Supplementary Table 10. Linear mixed model results for the medial prefrontal cortex (mPFC) response.

Fixed Effects					
	Estimate	SE	95% CI	t	p
Intercept	0.79	0.21	0.39 – 1.20	3.85	.0002
Nat vs. Urb	0.08	0.24	-0.40 – 0.57	0.33	.739
Nat vs. Ind	0.10	0.24	-0.38 – 0.59	0.43	.670

Random Effects		
	Variance	SD
Participant (Intercept)	0.611	0.78

Model fit		
R^2	Marginal	Conditional
	0.001	0.29

Note: p-values for fixed effects calculated using Satterthwaites approximations. All p-values are based on two-sided tests (uncorrected). Confidence Intervals (CI) have been calculated using the Wald method. SE = standard error, SD = standard deviation. Model equation: mPFC_Response ~ Environment + (1|Participant). Nat = Nature, Urb = Urban, Ind = Indoor. Source data are provided as a Source Data file.

Supplementary Table 11. Linear mixed model results for the periaqueductal grey (PAG) response.

Fixed Effects					
	Estimate	SE	95% CI	t	p
Intercept	0.53	0.10	0.34 – 0.73	5.31	<.000001
Nat vs. Urb	0.09	0.12	-0.14 – 0.33	0.77	.443
Nat vs. Ind	0.03	0.12	-0.21 – 0.27	0.25	.807

Random Effects		
	Variance	SD
Participant (Intercept)	0.148	0.38

Model fit		
R^2	Marginal	Conditional
	0.003	0.30

Note: p-values for fixed effects calculated using Satterthwaites approximations. All p-values are based on two-sided tests (uncorrected). Confidence Intervals (CI) have been calculated using the Wald method. SE = standard error, SD = standard deviation. Model equation: PAG_Response ~ Environment + (1|Participant). Nat = Nature, Urb = Urban, Ind = Indoor. Source data are provided as a Source Data file.

Supplementary Table 12. Linear mixed model results for the posterior insula (pINS) response.

Fixed Effects	Estimate	SE	95% CI	t	p
Intercept	0.36	0.13	0.11 – 0.61	2.81	.006
Nat vs. Urb	0.97	0.16	0.65 – 1.28	6.10	<.000001
Nat vs. Ind	0.55	0.21	0.14 – 0.95	2.65	.010
Left vs. Right	0.28	0.08	0.12 – 0.45	3.39	.0009
Nat vs. Urb * Left vs. Right	-0.12	0.11	-0.33 - 0.10	-1.08	.281
Nat vs. Ind * Left vs. Right	-0.09	0.11	-0.10 – 0.11	-0.92	.357

Random Effects	Variance	SD	Correlation		
Participant (Intercept)	0.644	0.803			
Nat vs. Urb (Slope)	0.943	0.971	-.46		
Nat vs. Ind (Slope)	1.800	1.342	-.42	.35	
Left vs. Right (Slope)	0.055	0.233	.42	.15	.24

Model fit	Marginal	Conditional
R^2	0.10	0.90

Note: p-values for fixed effects calculated using Satterthwaites approximations. All p-values are based on two-sided tests (uncorrected). Confidence Intervals (CI) have been calculated using the Wald method. SE = standard error, SD = standard deviation. Model equation: pINS_Response ~ Environment*Hemisphere + (1+Environment+Hemisphere|Participant). Nat = Nature, Urb = Urban, Ind = Indoor. Source data are provided as a Source Data file.

Supplementary Table 13. Linear mixed model results for the primary somatosensory cortex (S1) response.

Fixed Effects					
	Estimate	SE	95% CI	t	p
Intercept	-0.15	0.16	-0.47 – 0.16	-0.95	.346
Nat vs. Urb	0.33	0.18	-0.03 – 0.68	1.79	.075
Nat vs. Ind	0.21	0.22	-0.21 – 0.64	0.92	.361
Left vs. Right	0.68	0.14	0.39 – 0.96	4.68	.000006
Nat vs. Urb * Left vs. Right	-0.05	0.19	-0.43 – 0.33	-0.25	.798
Nat vs. Ind * Left vs. Right	0.21	0.19	-0.17 – 0.59	1.09	.276

Random Effects					
	Variance	SD	Correlation		
Participant (Intercept)	0.811	0.900			
Nat vs. Urb (Slope)	0.686	0.828	-.28		
Nat vs. Ind (Slope)	1.520	1.232	-.39	.27	
Left vs. Right (Slope)	0.106	0.325	-.43	.98	.45

Model fit		
R^2	Marginal	Conditional
	0.09	0.75

Note: p-values for fixed effects calculated using Satterthwaites approximations. All p-values are based on two-sided tests (uncorrected). Confidence Intervals (CI) have been calculated using the Wald method. SE = standard error, SD = standard deviation. Model equation: S1_Response ~ Environment*Hemisphere + (1+Environment+Hemisphere|Participant). Nat = Nature, Urb = Urban, Ind = Indoor. Source data are provided as a Source Data file.

Supplementary Table 14. Linear mixed model results for the secondary somatosensory cortex (S2) response.

Fixed Effects					
	Estimate	SE	95% CI	t	p
Intercept	0.48	0.14	0.20 – 0.75	3.42	.001
Nat vs. Urb	0.59	0.13	0.34 – 0.85	4.57	.00002
Nat vs. Ind	0.58	0.17	0.24 – 0.93	3.36	.001
Left vs. Right	0.32	0.08	0.17 – 0.48	4.17	.00006
Nat vs. Urb * Left vs. Right	-0.25	0.09	-0.43 – -0.09	-2.98	.003
Nat vs. Ind * Left vs. Right	-0.19	0.09	-0.37 – -0.02	-2.22	.028

Random Effects					
	Variance	SD	Correlation		
Participant (Intercept)	0.865	0.930			
Nat vs. Urb (Slope)	0.635	0.797	-.52		
Nat vs. Ind (Slope)	1.297	1.139	-.37	.51	
Left vs. Right (Slope)	0.111	0.332	-.32	-.04	.00

Model fit		
R^2	Marginal	Conditional
	0.06	0.92

Note: p-values for fixed effects calculated using Satterthwaites approximations. All p-values are based on two-sided tests (uncorrected). Confidence Intervals (CI) have been calculated using the Wald method. SE = standard error, SD = standard deviation. Model equation: S2_Response ~ Environment*Hemisphere + (1+Environment+Hemisphere|Participant). Nat = Nature, Urb = Urban, Ind = Indoor. Source data are provided as a Source Data file.

Supplementary Table 15. Linear mixed model results for the superior parietal lobe (SPL) response.

Fixed Effects					
	Estimate	SE	95% CI	t	p
Intercept	0.47	0.25	-0.03 – 0.97	1.84	.071
Nat vs. Urb	0.53	0.31	-0.09 – 1.15	1.68	.096
Nat vs. Ind	0.91	0.38	0.17 – 1.66	2.31	.019
Left vs. Right	0.72	0.23	0.26 – 1.66	3.07	.002
Nat vs. Urb * Left vs. Right	0.01	0.26	-0.51 – 0.53	0.04	.965
Nat vs. Ind * Left vs. Right	0.13	0.26	-0.39 – 0.65	0.47	.634

Random Effects					
	Variance	SD	Correlation		
Participant (Intercept)	2.353	1.534			
Nat vs. Urb (Slope)	3.143	1.773	-.57		
Nat vs. Ind (Slope)	5.346	2.312	-.45	.47	
Left vs. Right (Slope)	0.964	0.982	.21	.08	.30

Model fit		
R^2	Marginal	Conditional
	0.06	0.84

Note: p-values for fixed effects calculated using Satterthwaites approximations. All p-values are based on two-sided tests (uncorrected). Confidence Intervals (CI) have been calculated using the Wald method. SE = standard error, SD = standard deviation. Model equation: SPL_Response ~ Environment*Hemisphere + (1+Environment+Hemisphere|Participant). Nat = Nature, Urb = Urban, Ind = Indoor. Source data are provided as a Source Data file.

Supplementary Table 16. Linear mixed model results for the thalamus response.

Fixed Effects					
	Estimate	SE	95% CI	t	p
Intercept	0.27	0.09	0.09 – 0.46	2.91	.005
Nat vs. Urb	0.32	0.11	0.10 – 0.54	2.86	.006
Nat vs. Ind	0.44	0.12	0.21 – 0.68	3.73	.0005
Left vs. Right	0.13	0.05	0.03 – 0.23	2.60	.010
Nat vs. Urb * Left vs. Right	-0.09	0.06	-0.21 – 0.03	-1.52	.131
Nat vs. Ind * Left vs. Right	-0.10	0.06	-0.22 – 0.02	-1.67	.095

Random Effects					
	Variance	SD	Correlation		
Participant (Intercept)	0.393	0.627			
Nat vs. Urb (Slope)	0.524	0.724	-.54		
Nat vs. Ind (Slope)	0.606	0.779	-.42	.59	
Left vs. Right (Slope)	0.029	0.172	-.05	.25	.32

Model fit		
R^2	Marginal	Conditional
	0.05	0.92

Note: p-values for fixed effects calculated using Satterthwaites approximations. All p-values are based on two-sided tests (uncorrected). Confidence Intervals (CI) have been calculated using the Wald method. SE = standard error, SD = standard deviation. Model equation: Thal_Response ~ Environment*Hemisphere + (1+Environment+Hemisphere|Participant). Nat = Nature, Urb = Urban, Ind = Indoor. Source data are provided as a Source Data file.

Supplementary Table 17. Likelihood ratio tests (LRT) comparing models with or without nature connectedness to explain self-reported and neural responses to pain.

Variable	Model	npar	AIC	BIC	χ^2	df	p
Immediate ratings	Excl. NCI	28	5449.9	5603.2	5.67	6	.461
	Incl. NCI	34	5456.3	5642.4			
Signature Response	Excl. NCI	13	757.6	805.5	6.32	6	.388
	Incl. NCI	19	763.3	833.3			
Thalamus Response	Excl. NCI	17	410.7	473.4	12.24	6	.057
	Incl. NCI	23	410.5	495.2			
S2 Response	Excl. NCI	17	621.7	684.4	7.13	6	.308
	Incl. NCI	23	626.6	711.3			
pINS Response	Excl. NCI	17	692.9	755.6	8.25	6	.219
	Incl. NCI	23	696.7	781.4			

Note: AIC = Akaike Information Criterion, BIC = Bayesian Information Criterion, df = degrees of freedom, NCI = nature connectedness index, npar = number of parameters in model, pINS = posterior insula, S2 = secondary somatosensory cortex. All p-values are based on two-sided tests. Source data are provided as a Source Data file.

Supplementary References

1. Faul, F., Erdfelder, E., Buchner, A. & Lang, A.-G. Statistical power analyses using G*Power 3.1: Tests for correlation and regression analyses. *Behav Res Methods* **41**, 1149–1160 (2009).
2. Chen, G., Adleman, N. E., Saad, Z. S., Leibenluft, E. & Cox, R. W. Applications of multivariate modeling to neuroimaging group analysis: A comprehensive alternative to univariate general linear model. *Neuroimage* **99**, 571–588 (2014).
3. Meteyard, L. & Davies, R. A. I. Best practice guidance for linear mixed-effects models in psychological science. *J Mem Lang* **112**, 104092 (2020).
4. Farzaneh, M. *et al.* Comparative effect of nature-based sounds intervention and headphones intervention on pain severity after cesarean section: A prospective double-blind randomized trial. *Anesth Pain Med* **9**, e67835 (2019).
5. Lechtzin, N. *et al.* A randomized trial of nature scenery and sounds versus urban scenery and sounds to reduce pain in adults undergoing bone marrow aspirate and biopsy. *J Altern Complement Med* **16**, 965–972 (2010).
6. Lee, D. *et al.* Can visual distraction decrease the dose of patient-controlled sedation required during colonoscopy? A prospective randomized controlled trial. *Endoscopy* **36**, 197–201 (2004).
7. Tse, M. M. Y., Ng, J. K. F., Chung, J. W. Y. & Wong, T. K. S. The effect of visual stimuli on pain threshold and tolerance. *J Clin Nurs* **11**, 462–469 (2002).
8. Tanja-Dijkstra, K. *et al.* The soothing sea: A virtual coastal walk can reduce experienced and recollected pain. *Environ Behav* **50**, 599–625 (2018).
9. Letzen, J. E. *et al.* Test-retest reliability of pain-related brain activity in healthy controls undergoing experimental thermal pain. *J Pain* **15**, 1008–1014 (2014).
10. Quiton, R. L., Keaser, M. L., Zhuo, J., Gullapalli, R. P. & Greenspan, J. D. Intersession reliability of fMRI activation for heat pain and motor tasks. *Neuroimage Clin* **5**, 309–321 (2014).
11. Upadhyay, J. *et al.* Test–retest reliability of evoked heat stimulation BOLD fMRI. *J Neurosci Methods* **253**, 38–46 (2015).
12. Barr, D. J., Levy, R., Scheepers, C. & Tily, H. J. Random effects structure for confirmatory hypothesis testing: Keep it maximal. *J Mem Lang* **68**, 255–278 (2013).
13. Botvinik-Nezer, R. *et al.* Placebo treatment affects brain systems related to affective and cognitive processes, but not nociceptive pain. *Nat Commun* **15**, 6017 (2024).
14. Han, X. *et al.* Effect sizes and test-retest reliability of the fMRI-based neurologic pain signature. *Neuroimage* **247**, 118844 (2022).
15. Wielgosz, J. *et al.* Neural signatures of pain modulation in short-term and long-term mindfulness training: A randomized active-control trial. *Am J Psychiatry* **179**, 758–767 (2022).
16. Brett, M., Anton, J.-L., Valabregue, R. & Poline, J.-B. Region of interest analysis using an SPM toolbox. *Neuroimage* **16**, 1140–1141 (2002).
17. McMahan, E. A. & Estes, D. The effect of contact with natural environments on positive and negative affect: A meta-analysis. *J Environ Psychol* **10**, 507–519 (2015).
18. Watson, D., Clark, L. A. & Tellegen, A. Development and validation of brief measures of positive and negative affect: The PANAS scales. *J Pers Soc Psychol* **54**, 1063–1070 (1988).
19. Van Gent, P., Farah, H., Van Nes, N. & Van Arem, B. HeartPy: A novel heart rate algorithm for the analysis of noisy signals. *Transport Res F-Traf* **66**, 368–378 (2019).
20. Martin, L. *et al.* Nature contact, nature connectedness and associations with health, wellbeing and pro-environmental behaviours. *J Environ Psychol* **68**, 101389 (2020).
21. Richardson, M. *et al.* A measure of nature connectedness for children and adults: Validation, performance, and insights. *Sustainability* **11**, 3250 (2019).
22. Egner, L. E., Sütterlin, S. & Calogiuri, G. Proposing a framework for the restorative effects of nature through conditioning: Conditioned restoration theory. *IJERPH* **17**, 6792 (2020).
23. Stehl, P. *et al.* From childhood blue space exposure to adult environmentalism: The role of nature connectedness and nature contact. *J Environ Psychol* **93**, 102225 (2024).
24. Hoeppli, M.-E. *et al.* Dissociation between individual differences in self-reported pain intensity and underlying fMRI brain activation. *Nat Commun* **13**, 3569 (2022).
25. Reddan, M. C. & Wager, T. D. Modeling pain using fMRI: From regions to biomarkers. *Neurosci Bull* **34**, 208–215 (2018).
26. Woo, C.-W. & Wager, T. D. What reliability can and cannot tell us about pain report and pain neuroimaging. *Pain* **157**, 511–513 (2016).
27. Iachini, T. *et al.* The experience of virtual reality: Are individual differences in mental imagery associated with sense of presence? *Cogn Process* **20**, 291–298 (2019).
28. Bushnell, M. C., Čeko, M. & Low, L. A. Cognitive and emotional control of pain and its disruption in chronic pain. *Nat Rev Neurosci* **14**, 502–511 (2013).
29. Rolls, E. T., Joliot, M. & Tzourio-Mazoyer, N. Implementation of a new parcellation of the orbitofrontal cortex in the automated anatomical labeling atlas. *Neuroimage* **122**, 1–5 (2015).
