# Supplementary material for: Nature exposure induces analgesic effects by acting on nociception-related neural processing
Source: Nat Commun. 2025 Mar 13;16:2037. doi: 10.1038/s41467-025-56870-2 (PMC11906725; doi:10.1038/s41467-025-56870-2)
Supplement: Supplementary file 2 — Reporting summary [file 41467_2025_56870_MOESM2_ESM.pdf]

nature portfolio

Corresponding author(s): Claus Lamm

Last updated by author(s): Dec 16, 2024

Reporting Summary

Nature Portfolio wishes to improve the reproducibility of the work that we publish. This form provides structure for consistency and transparency in reporting. For further information on Nature Portfolio policies, see our Editorial Policies and the Editorial Policy Checklist.

Statistics

For all statistical analyses, confirm that the following items are present in the figure legend, table legend, main text, or Methods section.

n/a	Confirmed	
☐	☒	The exact sample size (*n*) for each experimental group/condition, given as a discrete number and unit of measurement
☐	☒	A statement on whether measurements were taken from distinct samples or whether the same sample was measured repeatedly
☐	☒	The statistical test(s) used AND whether they are one- or two-sided<br>*Only common tests should be described solely by name; describe more complex techniques in the Methods section.*
☐	☒	A description of all covariates tested
☐	☒	A description of any assumptions or corrections, such as tests of normality and adjustment for multiple comparisons
☐	☒	A full description of the statistical parameters including central tendency (e.g. means) or other basic estimates (e.g. regression coefficient) AND variation (e.g. standard deviation) or associated estimates of uncertainty (e.g. confidence intervals)
☐	☒	For null hypothesis testing, the test statistic (e.g. *F*, *t*, *r*) with confidence intervals, effect sizes, degrees of freedom and *P* value noted<br>*Give P values as exact values whenever suitable.*
☒	☐	For Bayesian analysis, information on the choice of priors and Markov chain Monte Carlo settings
☒	☐	For hierarchical and complex designs, identification of the appropriate level for tests and full reporting of outcomes
☐	☒	Estimates of effect sizes (e.g. Cohen's *d*, Pearson's *r*), indicating how they were calculated

Our web collection on statistics for biologists contains articles on many of the points above.

Software and code

Policy information about availability of computer code

Data collection	For the presentation of audiovisual stimuli and subjective ratings, we used MATLAB R2021a (MathWorks, 2021) and Psychtoolbox (Psychophysics Toolbox) Version 3 (Brainard, 1997; Pelli, 1997; Kleiner et al, 2007).
Data analysis	For the linear mixed model analysis of the behavioral data, as well as the extracted region of interest and signature data, we used R (R Core Team, 2023) and the lme4 package (Bates et al., 2014).<br>For the MRI data, we used SPM12 (Wellcome Trust Centre for Neuroimaging, www.fil.ion.ucl.ac.uk/spm), the Marsbar toolbox (Brett et al., 2002), and the DARTEL suite (Ashburner, 2007) for Diffeomorphic Anatomical Registration Through Exponentiated Lie Algebra running on MATLAB 2021a (MathWorks, 2021). For extracting the multivoxel signatures of pain (NPS, SIIPS1) we used scripts created by the developers of these signatures, which were made available to us after personal enquiry. For the pulse rate data reported in the Supporting Information we used the Python Heartrate Analysis Toolkit heartpy (van Gent et al., 2019) running on Python 3.9.12. (Python Software Foundation, https://www.python.org/)

For manuscripts utilizing custom algorithms or software that are central to the research but not yet described in published literature, software must be made available to editors and reviewers. We strongly encourage code deposition in a community repository (e.g. GitHub). See the Nature Portfolio guidelines for submitting code & software for further information.

Data

Policy information about availability of data

All manuscripts must include a data availability statement. This statement should provide the following information, where applicable:

- Accession codes, unique identifiers, or web links for publicly available datasets
- A description of any restrictions on data availability
- For clinical datasets or third party data, please ensure that the statement adheres to our policy

Behavioral data, region of interest and multivariate signature data extracted from the fMRI signal time course, thresholded whole-brain maps comparing pain>no-pain in urban vs. nature and indoor vs. nature environments, as well as unthresholded statistical maps for the pain>no-pain contrast in each environment, are asccessible at https://osf.io/t8dqu/.

Research involving human participants, their data, or biological material

Policy information about studies with human participants or human data. See also policy information about sex, gender (identity/presentation), and sexual orientation and race, ethnicity and racism.

Reporting on sex and gender	The participants included in the analyses were 24 women and 25 men. We considered gender in the study design by aiming to include an equal number of male and female participants. The analyses were not conducted separately for male and female participants, and there were no hypotheses regarding gender-specific effects. Gender was determined based on self-report. The data shared on OSF includes each participant's gender https://osf.io/t8dqu/.
Reporting on race, ethnicity, or other socially relevant groupings	We do not report on race, ethnicity, or other socially relevant groupings. There were no hypotheses regarding the impact of such groupings.
Population characteristics	N = 49 participants, 24 female, 25 male, mean age 25.24 (SD = 2.79, range = 20 - 35)
Recruitment	Recruitment was based on a participant recruitment platform (Vienna CogSciHub: Study Participant Platform (SPP), based on the Hamburg Registration and Organization Online Tool (hroot; Bock et al., 2014)) with student and non-student volunteers being invited for compensated (at a rate of 10€/h) study participation. The study invite did neither create overly strong incentives to partake, nor did it create any possible biases in addressing specific subpopulations (such as e.g., persons who were more or less connected to nature). That the study would entail pain was communicated. As such, participants afraid of pain or showing exaggerated responses to it may have been less inclined to volunteer. We also excluded persons with existing or prior neurological or psychiatric conditions (defined as exclusion criteria approved by the ethics committee). This could impact on the generalizability of the results to these kinds of subpopulations, but it will not impact the results for the participants included (which were based on a within-subjects crossover design).
Ethics oversight	The study was approved by the Ethics Committee of the University of Vienna (EK-Nr. 00729).

Note that full information on the approval of the study protocol must also be provided in the manuscript.

Field-specific reporting

Please select the one below that is the best fit for your research. If you are not sure, read the appropriate sections before making your selection.

☐ Life sciences ☒ Behavioural & social sciences ☐ Ecological, evolutionary & environmental sciences

For a reference copy of the document with all sections, see nature.com/documents/nr-reporting-summary-flat.pdf

Behavioural & social sciences study design

All studies must disclose on these points even when the disclosure is negative.

Study description	We conducted a preregistered repeat-crossover functional magnetic resonance imaging experiment using a within-participant design. Participants underwent three conditions presented in a counterbalanced order. We collected quantitative subjective ratings of pain (intensity and unpleasantness), quantitative recollections of the experienced pain, ratings of positive and negative affect, the perceived level of immersion of the shown environments, as well as interindividual differences in nature connectedness. Furthermore, we quantitatively assessed neural responses to pain by contrasting painful over non-painful electrical shocks in the scanner. We used region of interest (ROI) and signature-based analyses to investigate changes in the neural response to pain. For the operationalizations of ROI and signature responses, see the section 'Magnetic Resonance Imaging' of this document.
Research sample	The sample consisted primarily of university students in Vienna. The mean age was 25.24 years (SD = 2.79, range = 20 – 35). Of the participants, 24 were female and 25 were male. The sample is a convenience sample and not representative regarding age or gender or other sociodemographic factors. All participants had to meet standard inclusion criteria for neuroimaging and pain studies. The inclusion criteria were chosen to ensure that all participants could safely participate in an MRI study and were not suffering from pre-existing conditions or undergoing treatments that could interfere with normal pain processing.

Sampling strategy	The sample of this study is a convenience sample. We conducted an a-priori power analysis, which yielded a planned sample size of N = 41 participants. This sample size was based on a power analysis conducted for a repeated measures ANOVA model using G*Power 3.1 (1). Although the preregistered and reported statistical models in this work are linear mixed models (LMM; 2, 3), the project was powered for repeated measures ANOVAs, which would have served as a fallback in case of convergence issues in the LMMs. Since, compared to repeated measures ANOVA, LMMs in most situations have a higher power, the estimated sample size from the analyses can be seen as a conservative estimate for the targeted LMMs. The power analysis was based on previous studies investigating the effect of different environmental stimuli on pain perception (4–7). The average of reported effect sizes comparing differences between nature stimuli to a complete absence of stimulation while experiencing pain was used (Cohen`s d = .65). Studies directly comparing natural to urban environments in the domain of pain research revealed an effect size of similar magnitude (Cohen's d = .71) as the average reported for the remaining studies. Using a type-I error probability of alpha = 0.05, a power of 1-beta = 0.8, an e = 0.34 for non-sphericity corrections, and conservative estimates for repeated measures correlation of r = 0.4 between consecutive pain measurements (8–10), we estimated a targeted sample size of N = 41 participants. Due to published studies possibly overestimating effect sizes due to publication/survivorship bias, we decided to use a larger sample size of N = 48. We oversampled during data collection by recruiting 5 additional participants, targeting a total of N=53, to account for potential exclusions and to ensure that we would not fall below the target sample size of N = 48. Since we ended up excluding 4 participants, we slightly exceeded our target sample size, resulting in a final sample of N = 49. - For references indicated here please see the supplementary material of the submitted manuscript.
Data collection	Subjective data were collected on a computer using numerical rating scales presented on the MRI-compatible computer screen visible via a backprojection mirror system mounted on the head coil, during and directly after the functional runs. Participants used the button box of the scanner to rate pain intensity and unpleasantness immediately after twelve out of 32 shocks in each environment. Directly after a functional run, several additional questions were rated regarding positive and negative affect, the level of perceived immersion, as well as how much being immersed in the last environment helped them distract themselves from or tolerate the painful events. Numerical rating scales were presented using Psychtoolbox in Matlab. In addition to the participants, two researchers were present to operate the MRI and conduct the experiment. They sat at the MRI console outside the scanner room and connected to it via the standard patient/participant monitoring window. The researchers could thus see the participant, but not vice versa. The stimulus material was mirrored on a separate computer monitor next to the scanner console for the researchers to view and monitor task compliance. This setup allowed the researchers to see the stimuli and responses at all times, but they were strictly instructed to only interfere in case of obvious problems (such as stimuli not playing properly) or non-compliance (such as participants showing no or erratic button box entries). The researchers were not blind to experimental condition or study hypotheses. Participants were recruited by means of advertisements on- and offline and using a database of interested study participants implemented at the University of Vienna (https://spp.cogsci.univie.ac.at/). Due to the open-ended nature of the recruitment documents, which did not explicitly hint at the hypotheses being tested in the study, the potential for self-selection bias is considered low. Given that participants were also characterized by varying levels of nature connectedness and previous nature contact, we are confident that there was no self-selection bias towards individuals with a high connection to nature. Thus, we believe that self-selection is unliekly to have impacted the results.
Timing	Data were collected from April 2022 until September 2022. There was no gap in the collection period.
Data exclusions	Out of a collected sample of N = 53 participants, four participants had to be excluded due to technical problems with the pain stimulator and the scanner.
Non-participation	No participants dropped out or declined participation.
Randomization	Each participant was exposed to all three environments. The order of the environments was counterbalanced, with each person randomly assigned to one of the six possible sequences, ensuring that each sequence occurred with equal frequency.

Reporting for specific materials, systems and methods

We require information from authors about some types of materials, experimental systems and methods used in many studies. Here, indicate whether each material, system or method listed is relevant to your study. If you are not sure if a list item applies to your research, read the appropriate section before selecting a response.

Materials & experimental systems

n/a	Involved in the study
☒	☐ Antibodies
☒	☐ Eukaryotic cell lines
☒	☐ Palaeontology and archaeology
☒	☐ Animals and other organisms
☒	☐ Clinical data
☒	☐ Dual use research of concern
☒	☐ Plants

Methods

n/a	Involved in the study
☒	☐ ChIP-seq
☒	☐ Flow cytometry
☐	☒ MRI-based neuroimaging

Plants

Seed stocks

Report on the source of all seed stocks or other plant material used. If applicable, state the seed stock centre and catalogue number. If plant specimens were collected from the field, describe the collection location, date and sampling procedures.

Novel plant genotypes

Describe the methods by which all novel plant genotypes were produced. This includes those generated by transgenic approaches, gene editing, chemical/radiation-based mutagenesis and hybridization. For transgenic lines, describe the transformation method, the number of independent lines analyzed and the generation upon which experiments were performed. For gene-edited lines, describe the editor used, the endogenous sequence targeted for editing, the targeting guide RNA sequence (if applicable) and how the editor was applied.

Authentication

Describe any authentication procedures for each seed stock used or novel genotype generated. Describe any experiments used to assess the effect of a mutation and, where applicable, how potential secondary effects (e.g. second site T-DNA insertions, mosiacism, off-target gene editing) were examined.

Magnetic resonance imaging

Experimental design

Design type

Task-based and event-related. The runs represent task-based functional MRI using a standard experimental electrical pain stimulation task including painful and non-painful trials. Each pain block presented the painful and non-painful trials in the same pseudorandomized order in an event-related fashion.

Design specifications

The experiment included three functional runs. During each run a specific environment was presented and participants received electrical shocks. Thirty-two electrical shocks (16 painful and 16 non-painful) were administered per run. Each trial started with a colored visual cue displayed for 2.000 ms that indicated the next shock's intensity (painful = red, non-painful = yellow). After a variable pause where the cue disappeared (jittered with 3.500 ± 1.500 ms), another visual cue was presented for 1.000 ms with the electrical stimulus being administered for 500 ms simultaneously. The second visual cue matched the first cue in shape and size but had a colored filling. Next, the cue and shock disappeared for a variable duration (jittered with 3.500 ± 1.500 ms). An additional intertrial interval of 2.000 ms separated all trials.

Behavioral performance measures

Twelve pain intensity and unpleasantness ratings were collected for each functional run. We analyzed the ratings for both painful and non-painful trials and checked whether the ratings for the painful and non-painful trials averaged around the targeted subjective pain level of 6 and 1 during the calibration phase, respectively.

Acquisition

Imaging type(s)

Functional and structural

Field strength

3T

Sequence & imaging parameters

Each run acquired a separate functional volume using a multiband-accelerated gradient echo echoplanar imaging sequence, for one of the three pain blocks using the following parameters: Repetition time (TR) = 800 ms, echo time (TE) = 34 ms, flip angle = 50°, field of view (FOV) = 210 x 210 x 138 mm3, multi-band acceleration factor = 4, interleaved multi-slice mode, interleaved acquisition, matrix size = 96 × 96 x 36, voxel size = 2.18 × 2.18 × 3.84 mm3, 36 axial slices of the whole brain with slice thickness = 3.50 mm and an interslice gap of 0.34 mm. We used a magnetization-prepared rapid acquisition gradient echo sequence with the following parameters to obtain the structural image at the end of each scanning session: TR = 2,300 ms, TE = 2.29 ms, flip angle = 8°, FOV = 165 x 240 x 240 mm3, ascending acquisition, single shot multi-slice mode, 176 sagittal slices, matrix size = 176 x 256 x 256, voxel size = 0.94 × 0.935 × 0.935 mm3, slice thickness = 0.94 mm. Furthermore, field map images were acquired using a dual-echo gradient echo sequence to correct the functional images for magnetic field inhomogeneities, with the following parameters: TR = 400 ms, TE1 = 4.92 ms, TE2 = 7.38 ms, flip angle = 60°, FOV = 220 x 220 x 138 mm3, matrix size = 128 x 128 x 36, voxel size = 1.72 x 1.72 x 3.84 mm3, 36 axial slices aligned with the orientation of the functional images, and slice thickness = 3.84 mm.

Area of acquisition

Whole brain

Diffusion MRI ☐ Used ☒ Not used

Preprocessing

Preprocessing software

Preprocessing of the fMRI data was performed using SPM12 (Wellcome Trust Centre for Neuroimaging, www.fil.ion.ucl.ac.uk/spm) running on MATLAB 2021a (Mathworks, 2021), including the following steps: realignment and unwarping using participant-specific field maps, slice-time correction with the center slice as reference, coregistration of functional and structural images, segmentation into three tissue types (gray matter, white matter, cerebrospinal fluid), spatial normalization, and spatial smoothing with a 6-mm full-width at half maximum 3D Gaussian Kernel.

Normalization

Spatial normalization to Montreal Neurological Institute space using Diffeomorphic Anatomical Registration Through Exponentiated Lie Algebra (DARTEL); non-linear; using T1-weighted structural and segemented grey and white matter images per participant.

Normalization template

DARTEL and MNI152

Noise and artifact removal

Each design-matrix included six nuisance regressors from the realignment step accounting for movement-induced noise (six motion parameters representing rotation and translation into three directions).

Volume censoring

N/A

Statistical modeling & inference

Model type and settings

The first-level analyses performed on the whole brain level followed a general linear model (GLM) approach. A design matrix was specified in which the anticipation of painful and non-painful trials, the delivery of painful and non-painful trials as well as the ratings were modeled as experimental regressors per environment (i.e., run). The experimental regressors were time-locked to the onset of each respective trial phase and convolved using SPM12's standard hemodynamic response function in an event-related fashion. We computed the activity in each region of interest by extracting the mean percent signal change for the pain>no-pain first-level contrast using MarsBar toolbox for each environment. Furthermore, we computed the activity of each multivariate pain signature (NPS, SIIPS1) for each environment using scripts provided by the developers of these signatures. Both ROI and signature values are represented as continuous scores.

Effect(s) tested

We used the continuous scores for each ROI and MVPA signature in linear mixed effects models. We tested the main or interaction effect of the factor environment (three levels).

Specify type of analysis: ☐ Whole brain ☐ ROI-based ☒ Both

Anatomical location(s)

We created the following preregistered set of sphere-based ROIs (center [± x, y, z]; sphere size): amygdala (±20, -12, -10]; 10mm), anterior midcingulate cortex (aMCC; [-2, 23, 40], 10mm), anterior insula (aINS; [±33, 18, 6]; 10mm), posterior insula (pINS; [±44, -15, 4]; 10mm), medial prefrontal cortex (mPFC; [7, 44, 19]; 10mm), primary somatosensory cortex (S1; [±39, -30, 51]; 10mm), secondary somatosensory cortex (S2; [±39, -15, 18]; 10mm), periaqueductal gray (PAG; [0, -32, -10]; 6mm), superior parietal lobe (SPL; [±18, -50, 70]; 10mm), and thalamus ([±12, -18, 3]; 6mm).

Statistic type for inference

(See Eklund et al. 2016)

Inferences were based on the continuous scores for each ROI and pain signature. Depending on the type of analysis: MVPA signature based analyses: continuous score for NPS and SIIPS1 signature; ROIs: continuous score representing mean ROI values; whole-brain complementary: voxel-wise FWE-corrected using random Gaussian field theory, as implemented in SPM12

Correction

fMRI analyses including more that one dependent variable used familywise-error (FWE) correction (using random Gaussian field theory) at voxel-level ($p < .05$). The p-values of the pairwise comparisons from the ROI analysis were Bonferroni-Holm corrected (separated by the different descending modulatory (attention vs. emotion) and ascending pain circuits).

Models & analysis

n/a	Involved in the study
☒	☐ Functional and/or effective connectivity
☒	☐ Graph analysis
☐	☒ Multivariate modeling or predictive analysis

Multivariate modeling and predictive analysis

We used a-priori developed and independently determined signatures for pain processing that had been previously established using multivariate modeling. The extraction of each signature response was done using custom scripts provided to us by the developers of these signatures (Tor Wager et al.).
